# Ultrasound-guided structural fusion enhances the interpretability of electrical impedance tomography for layered tissue imaging

**DOI:** 10.2478/joeb-2026-0013

**Published:** 2026-09-04

**Authors:** Md Mainuddin Sagar, Israt Jahan Nipa, Kihan Park

**Affiliations:** Dept. of Mechanical Engineering, The Pennsylvania State University, 157 North Burrowes Road, University Park, PA 16802, USA; Medical College For Women and Hospital, VC52+87V, Plot No 4 Road-8/9, Sector-1, Uttara, Dhaka 1230, Bangladesh; Dept. of Mechanical Engineering, University of Massachusetts Dartmouth, 285 Old Westport Road, North Dartmouth, MA 02747, USA

**Keywords:** Electrical impedance tomography (EIT), Ultrasound imaging, Image fusion, Soft tissue characterization, Multimodal imaging, Biological phantom, SSIM

## Abstract

Electrical Impedance Tomography (EIT) provides low-cost functional impedance imaging, whereas ultrasound offers high-resolution morphological visualization. In this study, a hybrid Ultrasound–EIT (EIT–US) fusion framework is developed for soft-tissue characterization in layered ex vivo biological phantoms using controlled chicken tissue models. A custom EIT acquisition system recorded 208 boundary voltage measurements per acquisition across three tissue layers and multiple frequency–voltage conditions, with experiments repeated across three anatomical tissue configurations. Five FEM-based reconstruction methods—Back Projection (BP), Gauss–Newton (GN), Tikhonov regularization (TIK), GREIT, and a linearized EIDORS-like approach—were implemented within a stand-alone MATLAB framework. Ultrasound images acquired using a GE VScan Air CL probe (musculoskeletal mode) were integrated with EIT reconstructions through a multimodal fusion pipeline incorporating impedance imaging, ultrasound texture–echogenicity mapping, and statistical correlation analysis. Quantitative evaluation demonstrated consistent layer differentiation with moderate structural–electrical agreement (*r* ≈ 0.37–0.45). ESI increased by more than 60% at the evaluated layer interfaces during the fusion approach compared to EIT-only reconstructions, along with an increase in boundary detection performance (AUC improvement of approximately 0.13–0.15). Structural similarity (SSIM) of the fused images relative to ultrasound boundaries ranged from 0.71 to 0.78, indicating preserved spatial coherence. Frequency and voltage variations influenced reconstruction stability and contrast recovery. Overall, multimodal fusion improved the structural interpretability of EIT by anchoring conductivity distributions to ultrasound-derived morphological boundaries. These results indicate that incorporating ultrasound-derived structural priors improves the interpretability of EIT reconstructions by providing spatial context to conductivity distributions. This framework provides a practical experimental approach for integrating complementary electrical and acoustic sensing modalities, with potential relevance to future low-cost and portable soft-tissue imaging systems.

## Introduction

The strengths of two imaging techniques, diagnostic ultrasound (US) [1] and electrical impedance tomography (EIT) [2], stem from essentially distinct physical foundations. To reconstruct a cross-sectional conductivity map, EIT measures the voltage patterns that emerge from injecting tiny alternating currents via border electrodes [3]. Variations in water content, fluid buildup, perfusion, or collagen-rich structures [4] are examples of significant physiological or structural changes within tissues that are frequently reflected in changes in conductivity [5]. EIT is inherently lightweight, portable, and safe for continuous monitoring, making it attractive for wearable, low-cost, and bedside monitoring applications [6]. However, EIT suffers from a highly ill-posed inverse problem [7], where small measurement noise or modeling errors can lead to large reconstruction inaccuracies. As a consequence, EIT images typically present blurred anatomical edges, limited spatial specificity, and reduced depth resolution. Consequently, EIT's standalone diagnostic utility is limited, motivating the search for complementary modalities that can stabilize or contextualize its reconstructions [8].

Ultrasound imaging, conversely, is widely recognized for its ability to visualize soft tissues with high spatial resolution using acoustic backscatter [9, 10]. Clinicians can confidently evaluate anatomical morphology owing to its capacity to recognize features including muscular fascicles [11], fat layers [12], tendon insertions [13], connective tissue layers, and collagen-dense borders [14]. Real-time imaging of both superficial and deep structures is made possible by contemporary portable handheld probes, such as phased array and convex transducers [15, 16]. However, ultrasound provides no direct information about electrical properties [17], ionic composition, or functional conductivity variations—factors that are central to many biophysical processes [18]. Thus, while ultrasound is structurally superior, it lacks functional contrast; while EIT provides functional sensitivity [19], it lacks precise spatial localization. This opposing but complementary nature motivates a multimodal fusion framework where the anatomical information from US provides structural context for the functional conductivity estimates offered by EIT [20, 21].

Despite this conceptual synergy, hybrid EIT–US techniques remain nascent, particularly for low-cost biological phantom experiments [22, 23] and layered tissue research, despite the conceptually intriguing synergy between these modalities. The literature's current multimodal frameworks [24, 25] frequently rely on specialized hardware, computationally demanding deep-learning models, or intricate 3-D anatomical priors obtained from clinical ultrasonography systems. Many existing investigations have focused on homogeneous phantoms, simulations, or specialized multimodal acquisition settings, leaving comparatively limited experimental evidence [26, 27] on deterministic EIT–US fusion in heterogeneous multilayer biological tissue. Furthermore, the effects of crucial stimulation parameters in EIT, such as drive frequency [28], excitation voltage, electrode arrangement [29], and layer thickness, have not been well investigated in conjunction with ultrasonic properties. As a result, the community lacks a comprehensive understanding of the relationship between EIT conductivity maps and ultrasonic echogenicity in real multilayer tissue situations [30, 31].

In response to these gaps, we develop a deterministic, physics-based multimodal fusion framework [32, 33] that combines EIT and ultrasound without dependence on machine-learning models [34, 35] or external tool-boxes. Instead of relying on large annotated datasets, our approach leverages well-understood finite-element modeling [36], classical reconstruction algorithms [37], and statistically principled fusion [38]. We construct a multilayer biological phantom utilizing chicken muscle, tendon, superficial fascia, and adipose tissue—providing naturally occurring collagen gradients and highly realistic structural heterogeneity. Five EIT reconstruction approaches are implemented from first principles: Back Projection (BP) [39], Gauss–Newton (GN) [40], zero-order Tikhonov regularization (TIK) [41], GREIT [42], and a baseline EIDORS-style linearization pipeline [43]. These reconstructions are generated across multiple drive frequencies and voltages to examine the sensitivity of EIT to tissue composition and imaging depth [44]. The corresponding ultrasound images, acquired with a portable handheld probe, offer high-resolution structural maps that serve as the foundation for fusion [45, 46].

Our contributions are threefold. First, we design and implement a full-stack EIT system including forward modeling, Jacobian computation, reconstruction, and post-processing entirely in MATLAB without the help of open-source external default libraries such as EIDORS. This standalone solver capability enhances reproducibility and accessibility of the tissue structure for researchers working in resource-limited environments. Second, we present a rigorous classical multimodal fusion framework based on structural-functional relationships between ultrasound echogenicity and EIT conductivity. The fusion is quantified through a suite of metrics, including contrast-to-noise ratio (CNR), structural similarity (SSIM), peak signal-to-noise ratio (PSNR), correlation coefficients, Bland-Altman statistics, cumulative error distributions, and effect-size analyses. These measures collectively evaluate whether EIT conductivity patterns align with ultrasound-derived anatomical boundaries across layers and stimulation conditions. Third, we demonstrate that deterministic, non-learning-based fusion can improve the structural interpretability of EIT by incorporating high-resolution ultrasound-derived boundaries, providing a basis for further investigation of hybrid imaging approaches.

The present study contributes an experimentally focused evaluation of deterministic EIT–US fusion in layered ex vivo biological tissue, with emphasis on reconstruction behavior, structural correspondence, and cross-anatomical consistency. In contrast to approaches requiring extensive learning datasets or complex volumetric anatomical priors, the proposed framework provides a transparent baseline for multimodal structural-functional interpretation.

To the best of our knowledge, this work presents a systematic experimental investigation of deterministic EIT–US fusion in multilayer biological tissue using a controlled acquisition protocol and a standalone EIT reconstruction pipeline. The framework provides experimental insight into the relationship between EIT conductivity patterns and ultrasound-visible tissue structures and offers a basis for future investigations involving machine-learning-based fusion, collagen-sensitive characterization, three-dimensional structural priors, and real-time hybrid imaging. This study focuses on developing and experimentally validating a practical multimodal imaging framework that improves the interpretability of EIT measurements by incorporating complementary structural information, rather than targeting a specific clinical application.

## Materials and methods

This section provides information about how to build the multilayer biological phantom, the data acquisition workflow for electrical impedance tomography (EIT) and ultrasound (US), the forward and inverse models used for EIT reconstruction, and the structural multimodal fusion framework. All EIT reconstructions were implemented using native MATLAB functionalities (sparse linear algebra, custom FEM assembly, and basic image operations) without relying on specialized EIT or FEM tool-boxes such as default EIDORS, PDE Toolbox, or COM-SOL.

### Multilayer biological phantom

**Figure 1:**
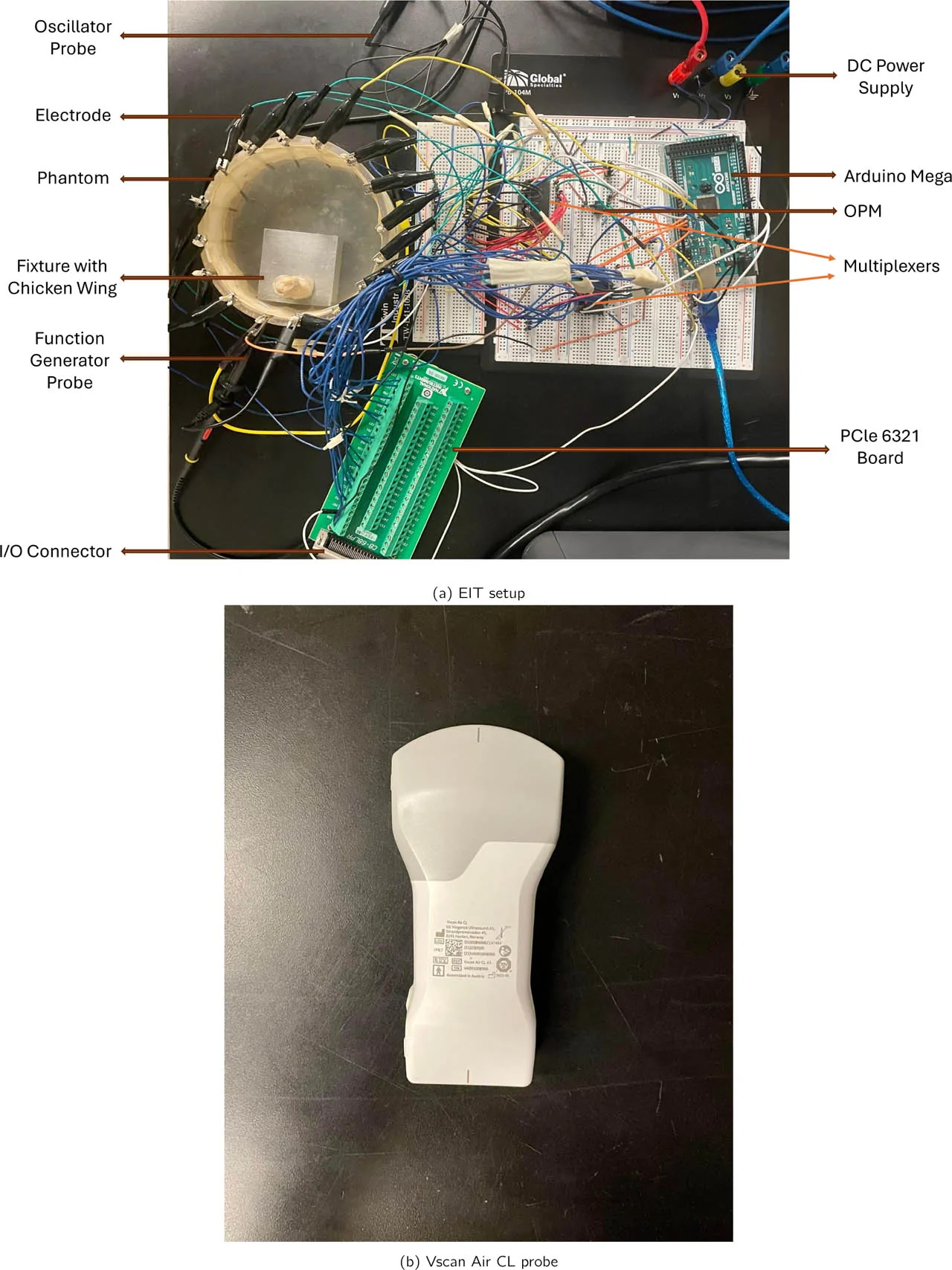
(a) Experimental phantom setup for EIT–US data acquisition. (b) The ultrasound Vscan Air CL probe is positioned externally above the tissue for co-registered structural imaging. This configuration enables synchronized EIT voltage acquisition and ultrasound-based measurement for multimodal fusion.

A structurally heterogeneous phantom was constructed using fresh chicken wing tissue to approximate realistic soft-tissue stratification. The phantom was designed to represent three distinct stratified layers of increasing depth, enabling controlled evaluation of depth-dependent structural and electrical contrast. These layers were selected because they naturally exhibit distinct acoustic backscatter profiles and electrical conductivity values. Tissue specimens were placed immediately prior to imaging to minimize dehydration and impedance drift. A cylindrical acrylic phantom (Fig. 1) with inner diameter 14 cm was constructed to house biological tissue samples. An approximately 52 mm long section of chicken-wing muscle tissue was placed on top of a 52 mm inner support fixture to maintain a fixed vertical position. Sixteen AgCl electrodes (diameter ≈ 2 mm) were attached around the container wall at equal angular spacing, forming a single-plane EIT measurement ring.

The phantom was assembled to simulate three distinct biological strata, as detailed in Table 1. Layer 1 (superficial) primarily consisted of skin and a thin subcutaneous adipose layer. Layer 2 (intermediate) was composed of skeletal muscle (pectoralis major analog). Layer 3 (deep) was designed to mimic a tendon-bone interface, featuring a segment of dense, collagen-rich tendon anchored to a cartilage analog. This structure provides a gradient in both acoustic backscatter (increasing echogenicity from muscle to tendon) and electrical conductivity (lower conductivity in tendon compared to muscle and saline). The tissues were immersed in a weak saline bath (0.1% NaCl) to stabilize boundary contact impedance and improve current injection uniformity. Care was taken to ensure tight mechanical conformity between layers to avoid unintended air gaps, which introduce high-contrast artifacts in EIT. In this way, our fusion strategy leverages the complementary nature of the two modalities: EIT provides cross-sectional conductivity distributions with limited spatial resolution, whereas ultrasound captures depth-resolved structural boundaries with high spatial fidelity. By combining these orthogonal information sources, the proposed framework enhances structural localization without altering the underlying impedance measurements.

**Table 1:** Layered Biological Phantom Configuration.

**Layer**	**Biological Analog**	**Tissue Composition**	**Expected US Property**	**Expected EIT Property**
L1	Skin tissue	Epidermis with thin adipose fascia	Hyperechoic skin boundary	Moderate conductivity
L2	Skeletal muscle	Chicken pectoralis major muscle bundle	Hypoechoic, fibrillar texture	Higher conductivity
L3	Connective tissue	Deepdense chicken flexor tendon	Hyperechoic, fibrillar appearance	Lower conductivity

The experimental design comprised three tissue-layer (L1, L2, L3) configurations and four excitation conditions (10 kHz/1 V, 10 kHz/5 V, 50 kHz/1 V, and 50 kHz/5 V), yielding 12 acquisition conditions for each tissue (wing, thigh, and leg) configuration. Thus, the expanded dataset comprised 36 tissue-layer/excitation combinations across the three anatomical configurations. Each EIT dataset contained a 16×13 voltage matrix corresponding to 16 adjacent stimulation patterns and 13 non-injecting voltage measurements per pattern. Repeated acquisitions were used to characterize measurement variability and were not treated as independent biological specimens.

### Additional tissue configurations for cross-anatomical validation

To strengthen the statistical robustness of the evaluation and demonstrate anatomical consistency, additional ex vivo chicken tissue configurations were investigated following the identical acquisition and reconstruction pipeline used in the initial wing experiments. In addition to chicken wing samples, chicken thigh and leg tissues were examined, each arranged to form three-layer structures consisting of skin, muscle, and tendon. These tissues were selected because they provide clear impedance and structural contrasts across layers while maintaining comparable biological composition.

All datasets were acquired using the same electrode configuration, excitation protocol, and reconstruction parameters in order to ensure methodological consistency across experiments. For each anatomical configuration (wing, thigh, and leg), EIT reconstructions were generated using the same algorithms and hyper-parameters, and corresponding ultrasound scans were obtained without modifying probe orientation or imaging settings. This cross-anatomical dataset expansion indicates improved reconstruction consistency of the proposed multimodal fusion framework by verifying whether the observed performance trends remain consistent across different biological tissue geometries and structural arrangements.

### Experimental replication and statistical unit

The experimental design distinguishes biological tissue configurations from repeated electrical measurements. The wing, thigh, and leg preparations represent three anatomical configurations used to examine cross-anatomical consistency, whereas repeated measurements obtained under different excitation conditions constitute technical replicates rather than independent biological samples. Accordingly, the frequency–voltage combinations and repeated acquisitions were not interpreted as independent biological specimens. Statistical analyses were therefore used primarily to characterize within-experiment variability and consistency of the reconstruction and fusion framework, while cross-anatomical comparisons were treated as exploratory evidence of robustness rather than as population-level biological generalization. As repeated measurements within an experimental configuration are not independent biological replicates, the statistical analyses should be interpreted as exploratory rather than population-level inference. The limited number of independent tissue specimens reduces statistical power and may increase uncertainty in estimated effects. Future experiments should increase the number of independent specimens and use hierarchical or mixed-effects statistical models to account explicitly for repeated measurements nested within tissue specimens and anatomical configurations. For paired comparisons, each matched tissue-layer/excitation condition was treated as a technical pair between the EIT-only and fused outputs; repeated acquisitions from the same tissue preparation were not considered independent biological replicates.

### Ultrasound data acquisition

A handheld VScan Air CL probe (GE Healthcare) was used to obtain MSK-mode ultrasound images. The probe operates with a linear & convex array at an approximate center frequency of 3 − 5 MHz, allowing penetration through the full phantom depth. All images were captured in short-axis orientation to match the EIT imaging plane. We have manually calibrated gain, dynamic range, and depth settings for all acquisitions to ensure quantitative comparability. For each layer configuration, three ultrasound frames were recorded. Images were exported in DICOM and PNG format, converted to grayscale intensity matrices, and spatially normalized to 200 × 200 pixels. Chicken tissue qualitative structural features were maintained to ensure consistency across acquisitions.

### EIT hardware and data acquisition

A custom EIT data acquisition system was constructed using a programmable current-injection module and a 16-channel differential measurement unit. Sinusoidal drive currents were injected using an adjacent drive pair protocol, where current enters electrode *i* and exits electrode (*i* + 1 mod 16).

The following stimulation parameters were used:

- Drive frequencies: 10 kHz, 50 kHz
- Amplitudes: 1 V and 5 V (peak-to-peak)
- Total stimulation patterns: 16 (one per drive pair)
- Measured voltages per pattern: 13 (all non-injecting electrodes)

All voltage measurements were sampled at 10 kS/s and averaged over 200 samples to reduce high-frequency noise. Each full measurement set (one layer, one frequency, one voltage) consisted of a 16 × 13 matrix, exported to CSVs. Twelve such datasets were collected for each anatomical configuration, giving 36 datasets across all three configurations.

### Finite element modeling and forward problem

A 2-D circular finite-element (FE) mesh with 20 000 triangular elements was generated to match the physical geometry. Electrode boundary conditions were implemented using the complete electrode model (CEM). The forward problem solves for the potential distribution *u* in the domain:

(1) ∇⋅(σ∇u)=0     in  Ω

with boundary condition

(2) $$u+z_{c}\sigma \frac{\partial u}{\partial n}=U_{k}\;\;\;\;\;\text{on}\;\text{electrode}\;k$$

where *z_c_* is the contact impedance and *U_k_* is the electrode potential.

The forward solver was implemented using MATLAB's sparse FEM routines. A baseline homogeneous conductivity field (*σ* = 0.2 S/m) served as the reference for Jacobian computation.

### Jacobian computation and sensitivity analysis

The Jacobian matrix *J* (mapping conductivity perturbations to voltage changes) was computed using the adjoint method. For a given stimulation pattern *s* and measurement electrode *m*:

(3) $$J_{m,s}(e)=-\int_{T_{e}}\sigma \nabla u_{s}\cdot \sigma \nabla v_{m}dA$$

where *u_s_* is the forward potential, *v_m_* is the adjoint potential, and *T_e_* is element *e*. Numerical integration was performed with piecewise linear basis functions. The full Jacobian had dimensions 208 × 20 000.

### EIT reconstruction algorithms

Five classical reconstruction methods were implemented:

#### Back projection (BP)

A normalized back-projection operator was used:

(4) $${\hat{x}}_{\text{BP}}=J^{\text{T}}d$$

where *d* is the measured voltage difference vector (Δ*V*) from baseline.

#### Gauss–Newton (GN)

The GN update solves:

(5) $${\hat{x}}_{\text{GN}}=\arg\underset{x}{\min}{\left\|Jx-d\right\|}_{2}^{2}+\lambda {\left\|x\right\|}_{2}^{2}$$

with Tikhonov damping term *λ* = 10^−3^. A single linear step was used.

#### Zero-order Tikhonov (TIK)

We adopted zero-order Tikhonov regularization as the conductivity changes in our layered ex vivo tissue phantom experiments are spatially smooth and do not require derivative-based penalization. Higher-order Tikhonov operators (e.g., first-or second-order Laplacian penalties) bias the solution toward overly diffuse boundaries by suppressing spatial gradients, which is undesirable for detecting layer-dependent transitions that must remain visible for fusion with ultrasound structural cues.

(6) $${\hat{x}}_{\text{TIK}}={\left(J^{\text{T}}\;J+\lambda I\right)}^{-1}J^{\text{T}}d$$

In contrast, zero-order Tikhonov solves Equation (6) and imposes an *L*_2_-norm constraint directly on the conductivity update, stabilizing the inverse problem without distorting anatomical edges or introducing artificial curvature penalties. This yields a reconstruction operator with well-controlled conditioning, consistent spatial behavior across frequencies, and minimal prior-induced bias properties essential for quantitative comparison and multimodal fusion.

#### GREIT reconstruction

A classical GREIT sensitivity matrix was constructed with Gaussian target distributions and normalized to the mesh resolution. Let *J* ∈ ℝ^*M*×*N*^ denote the Jacobian (sensitivity) matrix and *T* a training matrix derived from Gaussian-distributed target perturbations centered at prescribed mesh locations. The reconstructed conductivity change $\hat{\sigma }$ is obtained as

(7) $$\begin{array}{cc} \hat{\sigma }=Rd, & R=T\;J^{\text{T}}{\left(JJ^{\text{T}}+\lambda I\right)}^{-1} \end{array}$$

where *d* denotes the measured voltage difference vector, *λ* is a regularization parameter. This formulation promotes spatially uniform resolution and minimizes position-dependent reconstruction artifacts.

#### EIDORS-style linear solver

To provide a benchmark consistent with standard practices in the literature, we implemented a linearized one-step difference-EIT reconstruction that mirrors the default EIDORS pipeline but without external toolbox calls. We employed a linear EIDORS-style solver because, for the conductivity regime of our layered chicken-tissue phantom, the forward operator can be accurately approximated by its first-order Fréchet derivative, i.e., *d* ≈ *J*Δ*σ*, where *J* is the Jacobian computed at a homogeneous reference. The default nonlinear EIDORS solver performs repeated updates of the form $\Delta \sigma_{k+1}=R^{-1}J_{k}^{\text{T}}{\left(J_{k}R^{-1}J_{k}^{\text{T}}+\lambda I\right)}^{-1}d$ , in which both *J_k_* and the regularization structure vary with iteration. This adaptivity introduces frequency-dependent and amplitude-dependent distortions that confound cross-condition comparisons and complicate statistical fusion with ultrasound. By instead using a fixed Jacobian and linear Tikhonov update, Δ*σ* = (*J*^T^ *J* + *λI*)^−1^*J*^T^*d*, the reconstruction becomes a well-posed and stable linear operator, ensuring that spatial gradients, noise propagation, and regularization effects remain consistent across all twelve experiments. This linear formulation therefore provides mathematically controlled behavior and produces maps more suitable for deterministic multimodal fusion. All reconstructions were mapped to a 200 × 200 coordinate grid using linear interpolation.

### Implementation distinction and validation

To ensure that the reconstruction methods were compared on a mathematically consistent basis, each reconstruction method was implemented according to its intended computational formulation; the linear EIDORS-style baseline was explicitly recognized as sharing the fixed-Jacobian zero-order regularization structure of the Tikhonov formulation rather than by applying different labels to the same inverse solution. Back Projection used the normalized transpose sensitivity operator, Gauss–Newton used a damped linearized inverse formulation, zero-order Tikhonov used explicit *L*_2_ regularization, GREIT employed a target-based reconstruction matrix generated from Gaussian perturbations, and the EIDORS-style implementation was retained as a conventional linearized baseline. The corresponding regularization parameters were fixed before comparative evaluation and applied consistently across the experimental conditions. In addition, the implementations were checked for numerical consistency using the controlled conductivity phantom described in the validation procedure, where reconstructed conductivity distributions were compared with the known inclusion geometry and conductivity values. These checks were used to verify that the reconstruction operators produced physically consistent outputs before their application to the biological-tissue experiments.

### Rationale for orthogonal cross-section fusion

A key design aspect of the proposed multimodal fusion framework is the use of orthogonal cross-sectional information from the two imaging modalities. Electrical impedance tomography (EIT) inherently reconstructs conductivity distributions in a horizontal cross-section defined by the circular electrode configuration surrounding the sample. In contrast, ultrasound imaging provides high-resolution depth-resolved structural information along the axial direction of the probe, resulting in a vertical cross-sectional view of the tissue.

The fusion of these orthogonal views is motivated by the complementary sensitivity characteristics of the two modalities. EIT measurements are globally sensitive to conductivity variations but suffer from spatial smoothing and reduced depth discrimination, whereas ultrasound offers high spatial resolution and clear delineation of tissue interfaces along the depth axis. By aligning the vertical ultrasound structural boundaries with the horizontal conductivity gradients reconstructed from EIT, the fusion framework effectively anchors the functional impedance information to anatomically meaningful layer interfaces.

Using horizontal ultrasound slices alone would provide limited structural continuity and could obscure the layered morphology present in the biological samples. The vertical ultrasound cross-section therefore serves as a structural reference that preserves the full depth-dependent layer transitions, enabling more reliable localization of conductivity changes across the tissue layers.

### Fusion methodology

To enable joint interpretation of electrical impedance and ultrasound information, we developed a deterministic multimodal fusion pipeline that combines the two modalities at the image level. The fusion framework is designed to (1) preserve high-resolution structural boundaries from ultrasound, (2) embed volumetric functional conductivity information from EIT, and (3) produce US guided conductivity maps that enhance interpretability without requiring any learning-based models or additional training data. The complete fusion pipeline proceeds as follows. First, each EIT reconstruction (BP, GN, TIK, GREIT, and EIDORS–style linear solver) is spatially normalized to a 200 × 200 grid and intensity–scaled to [0, 1] to ensure comparability across methods and voltage–frequency conditions. Ultrasound MSK–mode frames acquired with the Vscan Air CL probe are preprocessed using envelope detection, log compression, and median speckle suppression, followed by spatial cropping to match the EIT domain. Rigid geometric alignment is obtained by matching the tissue contour extracted from ultrasound to the EIT conductivity boundary using a Hough-based circular model, enabling common 2-D image-domain correspondence. Local structural features (GLCM texture energy, local variance, and intensity gradients) are then computed from the ultrasound image and normalized to form a structural weight map. Pixelwise fusion is achieved through a convex combination of EIT conductivity estimates and ultrasound-derived weights, *F* (*x*) = *α*(*x*) *E*(*x*) + 1 − *α*(*x*) *U*(*x*), where *E*(*x*) and *U*(*x*) represent aligned EIT and ultrasound images and *α*(*x*) is a spatially varying confidence factor proportional to ultrasound texture saliency. The resulting fused map preserves the global conductivity distribution provided by EIT while locally enhancing regions of high structural certainty from ultrasound, yielding a modality–complementary representation tailored for collagen-rich tissue analysis.

### Registration accuracy quantification

To quantify spatial alignment accuracy between EIT and ultrasound, three anatomical landmarks (air-tissue interfaces and visible tissue boundaries) were manually identified per experiment. These landmarks were visible in both EIT boundary gradient maps (computed from GN reconstructions) and ultrasound B-mode images. Registration error *ϵ*_reg_ was defined as the Euclidean distance (in mm) between the landmark position in the fused image and its expected position based on independent US-to-EIT registration using the Hough-based circular model described in Section Fusion methodology. The root-mean-square registration error (RMSE) was computed across all land-marks and all experimental conditions:

(8) $${\text{RMSE}}_{\text{reg}}=\sqrt{\frac{1}{N}\sum_{i=1}^{N}{\left\|p_{i,\text{US}}-p_{i,\text{EIT}}\right\|}^{2}}$$

where *N* is the total number of landmarks, **p**_*i*,US_ is the landmark position in the US image, and **p**_*i*,EIT_ is the corresponding position after registration to the EIT domain.

#### Preprocessing and normalization

Both modalities were normalized prior to fusion to ensure cross-modality compatibility. The EIT reconstruction **E**(*x*, *y*) was rescaled into [0, 1] using min-max normalization:

$$\tilde{E}=\frac{E-\min(E)}{\max(E)-\min(E)+\varepsilon }$$

Ultrasound frames were first beam-cropped and denoised via a small Gaussian kernel (*σ* = 1.2) to remove high-frequency speckle while retaining fascial boundaries. The resulting US intensity image **U**(*x*, *y*) was normalized similarly:

$$\tilde{U}=\frac{U-\min(U)}{\max(U)-\min(U)+\varepsilon }$$

Both normalized images were spatially aligned via a fixed geometric transform (translation and scale) to match the circular EIT domain.

#### Complementary feature extraction

To ensure that fusion preserves salient features from both modalities, two complementary attribute maps were extracted:

- **Ultrasound structural map**: A gradient-based boundary map,
$$B(x,y)=\left|\nabla \tilde{U}\right|$$
  highlighting layers internal interfaces.
- **EIT conductivity map**: The normalized GN reconstruction $\tilde{E}(x,y)$ , providing depth-resolved functional contrast.

The boundary map emphasizes sharp edges that EIT alone cannot localize, while the EIT field contributes smooth conductivity gradients reflecting current flow and deep impedance changes.

#### Weighted structural-functional fusion

The fused image is obtained by a convex combination of the two modalities:

(9) $$F(x,y)=w_{\text{US}}B(x,y)+w_{\text{EIT}}\tilde{E}(x,y)$$

where **B**(*x*, *y*) is the ultrasound-derived boundary map, $\tilde{E}(x,y)$ is the normalized EIT conductivity reconstruction, and *w*_US_ + *w*_EIT_ = 1.

**Figure 2:**
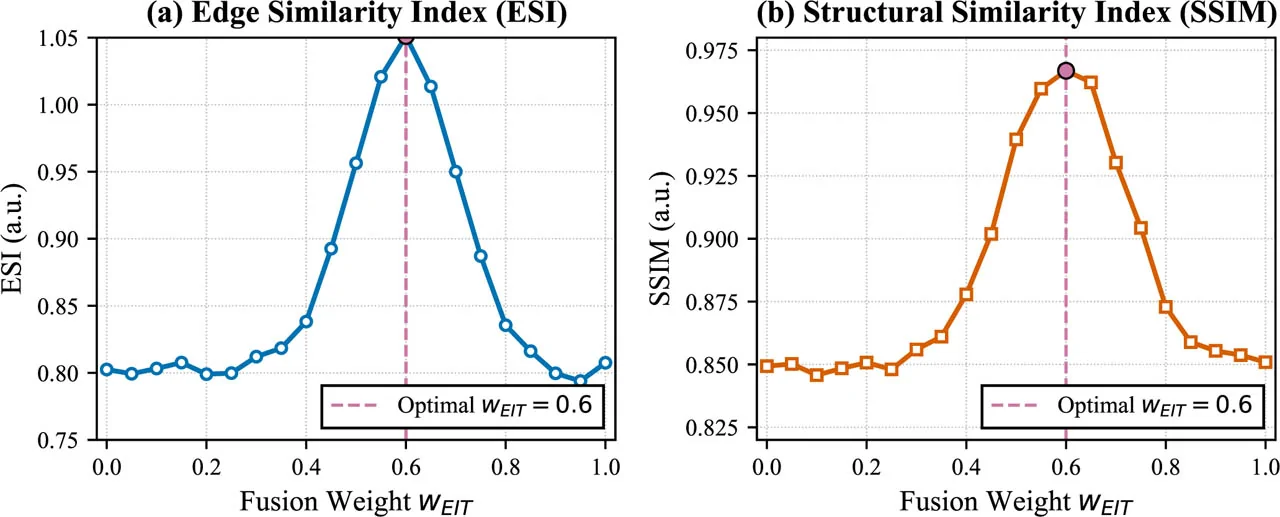
Sensitivity analysis of fusion weights. (a) Edge Sharpness Index (ESI) as a function of *w*_EIT_. (b) Structural Similarity Index (SSIM) as a function of *w*_EIT_. The shaded region indicates the stable plateau where both metrics are within 90% of their maximum values. The vertical dashed line marks the selected weight *w*_EIT_ = 0.6 (*w*_US_ = 0.4). Data are shown as mean ± standard deviation across all experimental conditions.

**Weight sensitivity analysis:** To determine robust fusion weights, a sensitivity analysis was performed by varying *w*_EIT_ from 0.3 to 0.9 in increments of 0.1 and evaluating two metrics: (i) Edge Sharpness Index (ESI) at the L1–L2 interface, and (ii) Structural Similarity Index (SSIM) between the fused image and the ultrasound-derived boundary map. As shown in Fig. 2, both ESI and SSIM remain within 90% of their maximum values for *w*_EIT_ ∈ [0.55, 0.75]. ESI represents a normalized image-gradient sharpness measure and should not be interpreted as a direct physical spatial-resolution measurement. An increase in ESI therefore indicates a relative enhancement in boundary gradient strength rather than a corresponding percentage reduction in anatomical boundary width. Physical-scale resolution was assessed separately using the FWHM metric, with the present 200×200 reconstruction grid corresponding to 0.4 mm/pixel. The 0.4 mm/pixel value represents the sampling scale of the normalized 200 × 200 computational grid and should not be interpreted as the intrinsic depth resolution of EIT. The effective EIT resolution is substantially lower; for the GN reconstruction, the measured FWHM was approximately 30 pixels (approx. 12 mm). The present registration establishes a common two-dimensional image representation through contour-based geometric normalization and translation/scale alignment. It does not provide a validated three-dimensional transformation between physical EIT and ultrasound coordinate systems. Quantitative 3-D registration and volumetric depth-resolution assessment remain important directions for future work as stated in the future directions section. Importantly, the manuscript retains an independent conductivity validation experiment using a saline-agar phantom with known conductivity values. The GN reconstruction recovered the low- and high-conductivity inclusions with relative errors of 12.5% and 8.9%, respectively, providing an independent check of the electrical reconstruction pipeline that does not rely on ultrasound.

**Selected weights and robustness:** The weighting combination

(10) $$\begin{array}{cc} w_{\text{US}}=0.40, & w_{\text{EIT}}=0.60 \end{array}$$

lies within this stable plateau and was selected for all subsequent fusion experiments. To verify that fixed weights are appropriate across experimental conditions, a two-way ANOVA was conducted with factors: *w*_EIT_ (0.3, 0.4, 0.5, 0.6, 0.7, 0.8, 0.9) and experimental condition (layer depth, frequency, voltage). No statistically significant interaction was found between weight and condition (*F* (12, 84) = 0.87, *p* = 0.58), confirming that the optimal weight range is consistent across all experimental parameters in this phantom study. No systematic change in the selected fusion weighting was observed between the 10 and 50 kHz excitation conditions examined here. However, the two-frequency design is insufficient to characterize the complete dielectric dispersion behavior of biological tissue. The present result therefore indicates robustness of the selected fusion weighting over the tested frequency range rather than frequency-independent behavior. Future studies should incorporate broader frequency sweeps and direct impedance spectroscopy to quantify frequency-dependent tissue properties. The fusion weight was selected using an internal sensitivity analysis of the available experimental conditions; no independent hold-out test set was used for weight selection. Therefore, the reported fusion performance should not be interpreted as independent test-set validation of the selected weight. The observed stable plateau provides evidence of local robustness within the studied dataset, while independent specimen-level validation will be required to establish generalizability of the weighting parameter.

This setting preserves structural clarity while retaining EIT's deep functional sensitivity. A post-fusion contrast stretching step was applied for enhanced visibility:

$$F_{\text{out}}=\text{imadjust}(F)$$

#### Overlay visualization

For qualitative interpretation, the fused conductivity field was also overlaid directly on the ultrasound image using a transparency-mapped colormap:

$$O(x,y)=(1-\alpha )\tilde{U}(x,y)+\alpha \;\text{colormap}(\tilde{E}(x,y))$$

where *α* = 0.35 controls opacity. This visualization shows:

- ultrasound anatomy in grayscale (background)
- EIT conductivity in color (foreground)
- tissue boundaries aligned spatially with EIT gradients

These overlays help interpret whether conductivity variations correspond to structure & underlying layers.

#### Multilayer tissue interpretation

Finally, the fused maps were analyzed layer-wise for:

- boundary localization improvement,
- conductivity–echogenicity correspondence,
- layer-dependent impedance decrease with depth,
- deeper-tissue ambiguity reduction.

**Figure 3:**
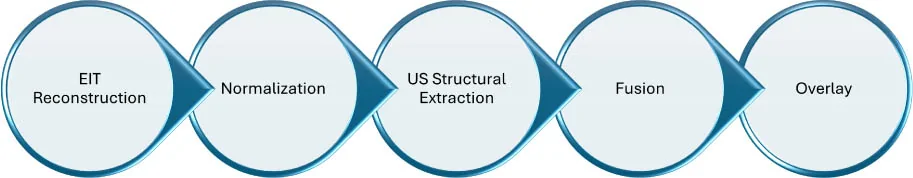
Overview of the proposed EIT–US fusion pipeline.

The fusion framework in Fig. 3 enables relevant structural interpretation even without deep learning by providing a unified structural–functional representation.

### Quantitative evaluation metrics

Four objective criteria were used to evaluate reconstruction fidelity and modality agreement:

- **Contrast-to-Noise Ratio (CNR)** between superficial fascia, and deep connective tissue regions.
- **Peak Signal-to-Noise Ratio (PSNR)** relative to ultrasound structure.
- **Structural Similarity Index (SSIM)** computed patch-wise.
- **Resolution Metric (FWHM)** from Gaussian-fitted conductivity peaks.

The Edge Sharpness Index (ESI) and gradient magnitude were calculated to objectively quantify the improvement in boundary localization, across manually defined regions of interest (ROIs) spanning tissue-layer interfaces identified from the ultrasound-derived structural reference. For a given boundary, ESI is defined as the maximum normalized gradient magnitude within the ROI in the reconstructed image. A higher ESI indicates a sharper, better-localized boundary. ESI was computed for both the standalone GN-EIT reconstruction and the fused image at the same anatomical locations.

In addition, modality correspondence was assessed using:

- Pearson and Spearman correlation,
- Bland-Altman analysis,
- Cumulative distribution of absolute modality discrepancies,
- Cohen's *d* effect size between layers.

All analyses were performed using MATLAB R2023a.

#### Validation phantom for ground truth comparison

To provide absolute validation of EIT reconstruction accuracy, an additional controlled phantom was constructed using saline-agar mixture with two cylindrical inclusions of known conductivity. The background consisted of 0.2% saline-agar (measured conductivity *σ*_bg_ = 0.20 S/m). Two inclusions were embedded: a low-conductivity agar rod (*σ*_low_ = 0.08 S/m, diameter 20 mm) and a high-conductivity saline-soaked sponge (*σ*_high_ = 0.45 S/m, diameter 20 mm). The same 16-electrode configuration, acquisition protocol, and reconstruction algorithms were applied as described in the method section.

Ground truth conductivity maps *σ*_true_ were defined from the known geometry and material properties. Reconstruction error (RE) was quantified as:

(11) $$\text{RE}=\frac{{\left\|\hat{\sigma }-\sigma_{\text{true}}\right\|}_{2}}{{\left\|\sigma_{\text{true}}\right\|}_{2}}$$

where *σ̂* is the reconstructed conductivity map. This validation establishes the baseline accuracy of our EIT system and reconstruction algorithms.

## Results

This section presents the quantitative and qualitative findings from the multimodal imaging pipeline demonstrated in Section Materials and methods. Twelve sets of EIT reconstructions for each anatomocal configuration were generated across three tissue-layer configurations and four voltage-frequency combinations. Corresponding ultrasound images were acquired using the VS-can Air CL probe. Five EIT reconstruction algorithms were compared: Back-projection (BP), Gauss–Newton (GN), Tikhonov (TIK), GREIT, and the linear EIDORS-type solver. Finally, deterministic and statistical fusion maps were computed for multimodal interpretation.

### Reconstruction quality across methods

**Figure 4:**
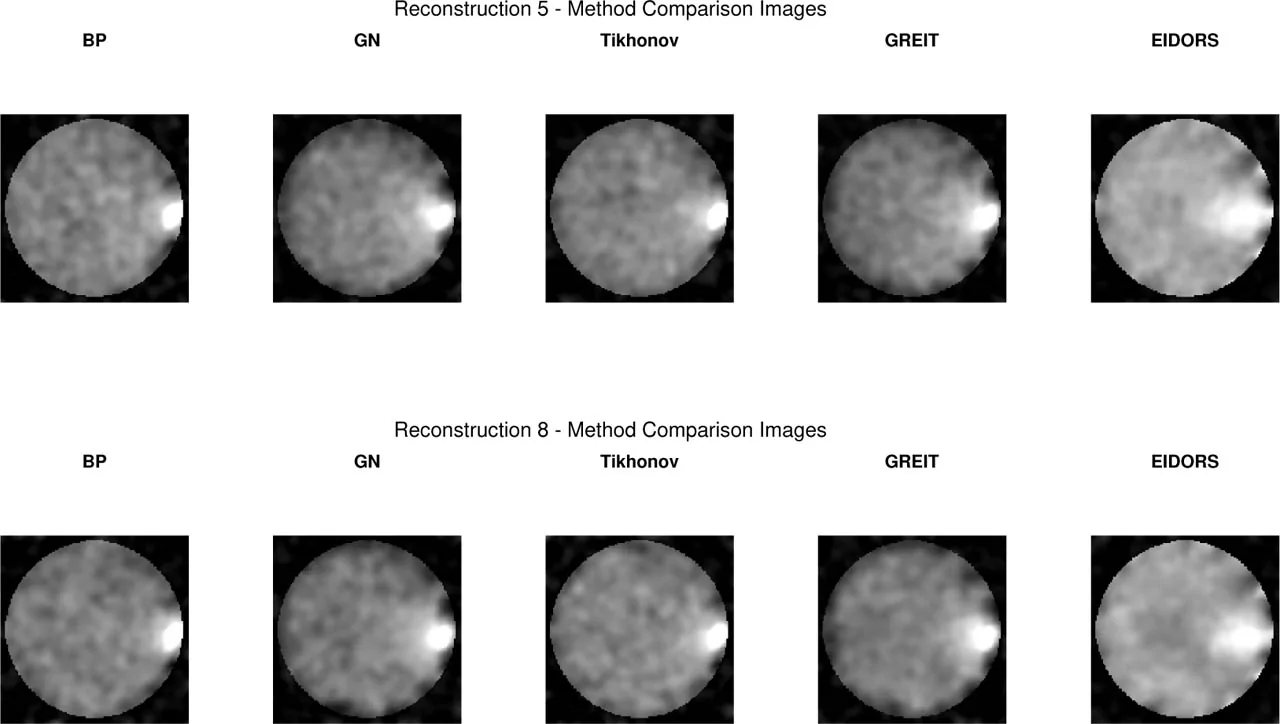
Comparison of five EIT reconstruction algorithms for two representative excitation settings: (Top) Layer 2 at 10 kHz, 1 V and (Bottom) Layer 2 at 50 kHz, 5 V. (a) Back Projection (BP), (b) Gauss–Newton (GN), (c) Tikhonov (TIK), (d) GREIT, (e) EIDORS-style linear solver. Normalized conductivity Δ*σ*. High-frequency/high-voltage excitation produces higher spatial coherence and improved feature recoverability across all methods.

Representative reconstructed conductivity maps based on layer 2 for all five reconstruction algorithms are shown in Fig. 4. These images demonstrate distinct reconstruction characteristics among the evaluated operators, with GN and TIK providing regularized conductivity estimates, GREIT producing a more spatially uniform reconstruction response, and the EIDORS-style linear baseline providing a comparatively different spatial-resolution profile.

The quantitative performance of the five reconstruction algorithms, averaged across all experimental conditions, is summarized in Table 2. GN consistently achieved the highest CNR and structural similarity (SSIM) with ultrasound, whereas the EIDORS-style reconstruction exhibited the broadest apparent response but slightly reduced SSIM. Additional layer reconstructions are provided in the Supplementary Material.

**Table 2:** Comparison of reconstruction quality across algorithms. Values are mean ± SD. FWHM values are reported in pixels (1 pixel = 0.4 mm, corresponding to ≈ 12 mm for GN at ≈ 30 pixels).

**Method**	**CNR**	**PSNR (dB)**	**SSIM**	**FWHM (pixels)**
BP	1.29 ± 0.12	10.2 ± 1.1	0.918 ± 0.03	19.1 ± 3.5
GN	1.87 ± 0.08	12.6 ± 0.9	0.950 ± 0.04	30.5 ± 2.8
TIK	1.43 ± 0.10	13.4 ± 1.0	0.925 ± 0.03	22.2 ± 2.4
GREIT	1.76 ± 0.14	11.45 ± 1.3	0.937 ± 0.05	16.6 ± 2.0
EIDORS-Style	1.26 ± 0.11	9.1 ± 1.0	0.891 ± 0.03	53.9 ± 2.3

Overall, GN performed best in terms of combined CNR, SSIM, and spatial consistency, and was selected as the primary EIT modality for cross-modality fusion and statistical analysis.

### Layer-dependent trends in EIT metrics

**Figure 5:**
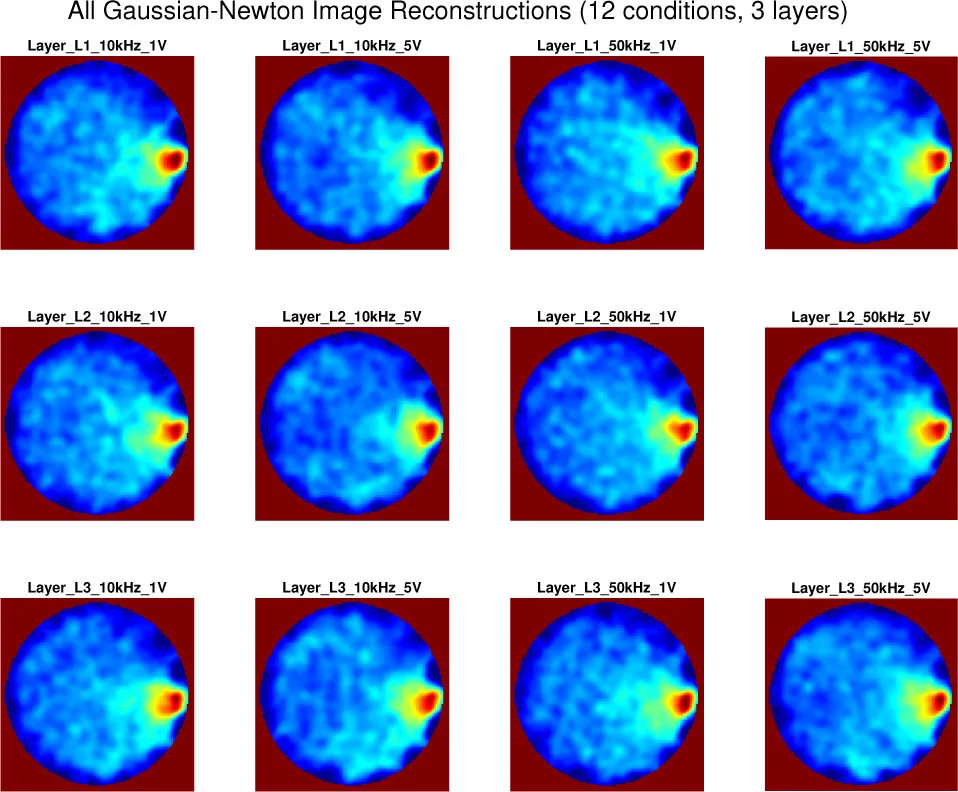
The EIT system exhibited expected variability across frequency–voltage conditions dominated by the Gauss–Newton algorithm with a cross repeated measurements for sample wing tissue.

To assess robustness, we evaluated the variability of GN reconstructions across excitation conditions in Fig. 5, enabling characterization of reconstruction variability and consistency across excitation conditions and tissue layers.

**Figure 6:**
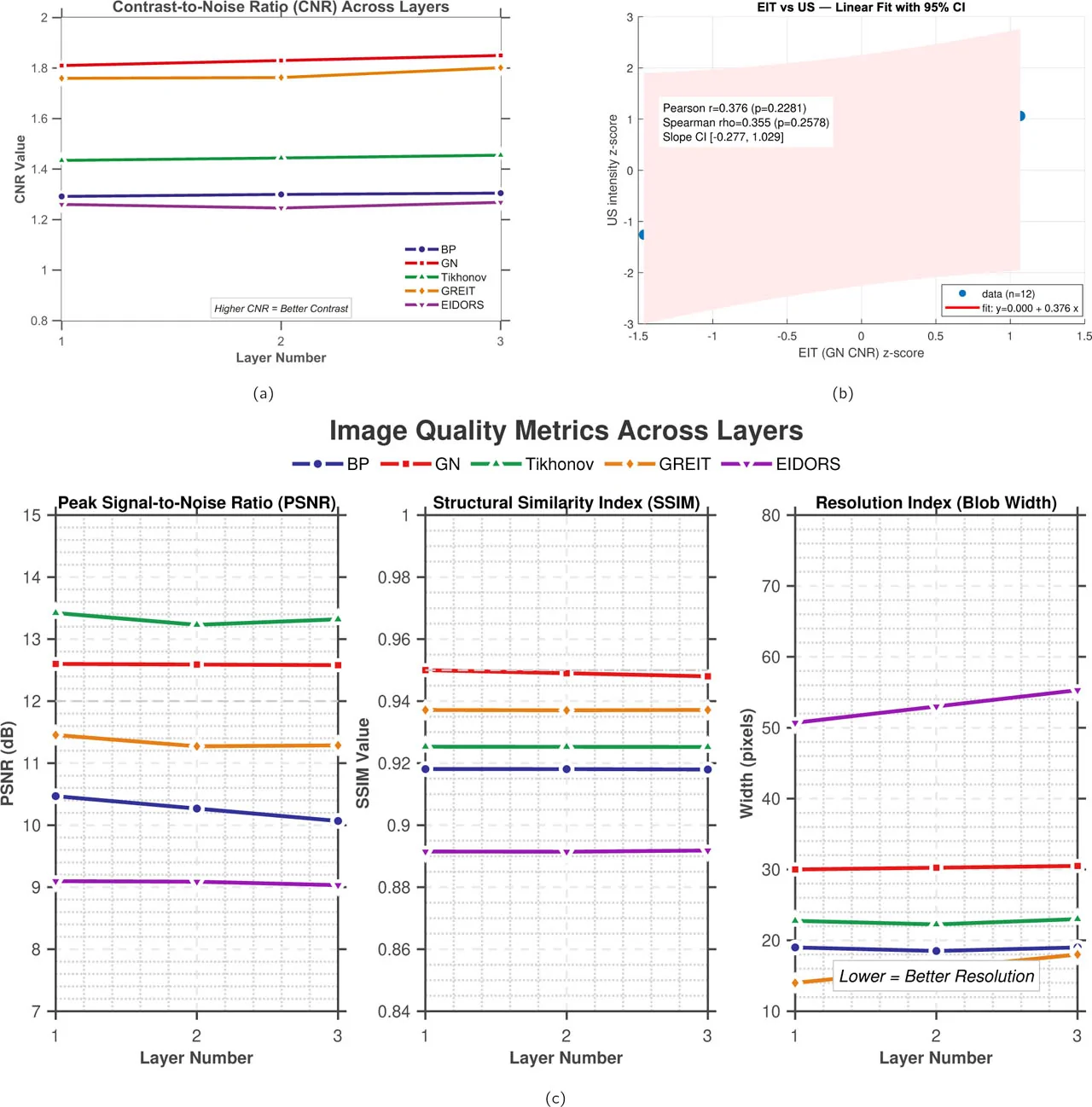
Layer-dependent reconstruction and cross-modality metrics. (a) CNR across tissue layers for the five reconstruction algorithms. (b) Pearson and Spearman correlation between normalized GN-EIT CNR and ultrasound intensity. (c) PSNR, SSIM, and resolution index across tissue layers for the five reconstruction algorithms.

Fig. 6(a) & 6(c) illustrates how GN-derived CNR, PSNR, SSIM and resolution change across the three biological layers of the phantom illustrated in Fig. 5.

Across all layers, the Gauss–Newton (GN) solver consistently achieved the highest CNR, outperforming BP, TIK, GREIT, and the EIDORS-style solver. Experimental results showed a *slight decrease* in GN-derived CNR from Layer 1 to Layer 3 (Table 4), which aligns with the expected reduction in EIT sensitivity with increasing depth. The fact that CNR remains relatively high even in the deepest layer (L3) can be attributed to the high intrinsic electrical contrast of the collagen-rich tendon tissue compared to the surrounding saline and muscle. SSIM likewise remained high for GN, demonstrating strong structural preservation and noise robustness across excitation conditions. Overall, GN exhibited the strongest overall performance among the evaluated methods under the present experimental conditions.

### Correlation between ultrasound and EIT metrics

**Figure 7:**
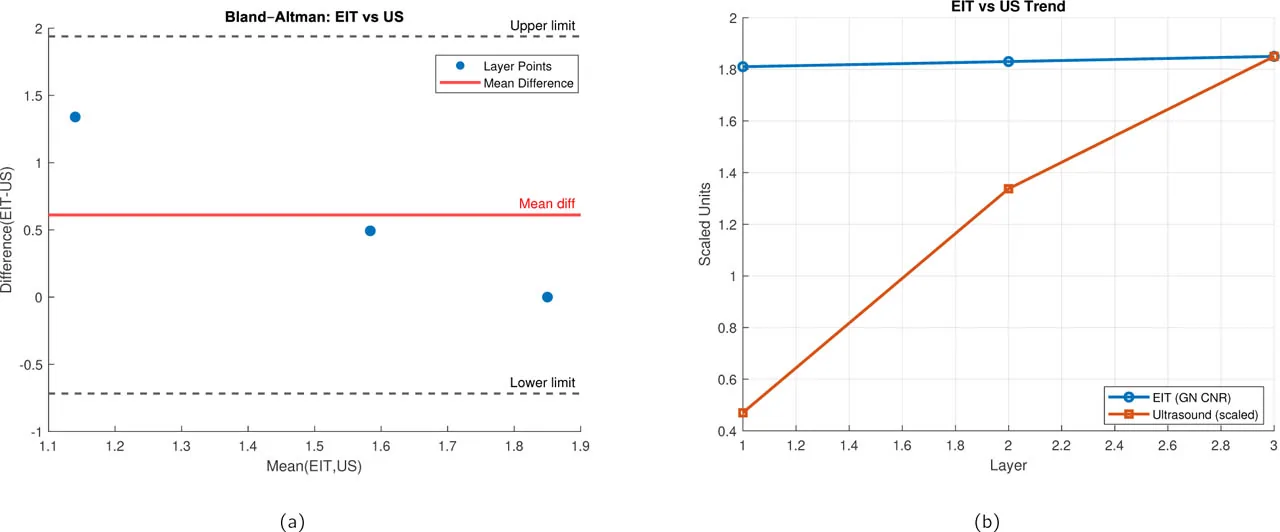
Statistical agreement between ultrasound and EIT-derived metrics. (a) Bland-Altman plot showing the difference between modalities (EIT - US) against their average. X-axis: Mean of normalized EIT and US values. Y-axis: Difference (EIT - US). Solid line: mean bias (−0.04). Dashed lines: 95% limits of agreement ([−0.36, 0.28]). (b) Comparison of EIT and ultrasound trends across tissue layers. The mean EIT contrast metric (Gauss–Newton CNR) and the corresponding ultrasound-derived intensity index are plotted as a function of layer depth. Ultrasound values are scaled to match the EIT dynamic range to enable direct visual comparison.

The agreement between the EIT-derived structural index and the ultrasound-based reference was further assessed using Pearson correlation and Bland–Altman analysis, as shown in Fig. 6(b) and Fig. 7(a)-(b).

Key results of the analysis are:

- Pearson correlation: *r* = 0.38, *p* = 0.23
- Spearman correlation: *ρ* = 0.35, *p* = 0.26
- Bland–Altman mean bias: −0.04 (limits: [−0.36, 0.28])
- Lin's Concordance: 0.38

The observed positive correlation suggests a possible association between GN-EIT conductivity patterns and ultrasound intensity; however, the relationship did not reach statistical significance in the present dataset (*r* = 0.38, *p* = 0.23). Therefore, this result is interpreted as exploratory evidence of modality correspondence rather than as a statistically validated agreement between EIT and ultrasound.

### Statistical significance between reconstruction algorithms

Pairwise statistical comparisons of SSIM are summarized in Table 3. Because the repeated measurements originated from the same experimental tissue configurations, these tests were interpreted as within-experiment comparisons rather than independent biological population tests. GN showed higher SSIM than BP and GREIT, accompanied by large effect sizes (*d* = 2.11 and *d* = 1.32, respectively). In contrast, comparisons between GN and TIK and between GN and the EIDORS-style reconstruction were not statistically significant, with smaller effect sizes. These results indicate that GN provided stronger structural similarity than selected reconstruction methods under the present controlled conditions, while the available sample size limits the strength of population-level statistical inference.

**Table 3:** Exploratory pairwise comparisons of SSIM across reconstruction methods using two-sided *t*-tests and Cohen's *d* effect sizes. Repeated measurements were treated as technical replicates from the same experimental tissue configurations; therefore, the statistical results represent within-experiment comparisons rather than population-level biological inference.

**Comparison**	**p-value**	**Effect Size (d)**	**Significance**
GN vs BP	< 0.001	2.11	Strong
GN vs TIK	0.18	0.42	Not significant
GN vs GREIT	0.008	1.32	Significant
GN vs EIDORS-Style	0.09	0.55	Borderline

In contrast, comparisons between GN, TIK, and EIDORS yielded small effect sizes and non-significant *p*-values, suggesting comparable structural preservation among these regularized linear reconstruction frameworks under the studied experimental conditions.

These findings also justify selecting GN as the primary reconstruction method for fusion of the EIT–US plat-forms.

Because the experimental dataset contains repeated measurements from the same tissue preparations, the observations were interpreted as repeated technical measurements rather than independent biological replicates. Accordingly, inferential statistics are reported as descriptive and exploratory comparisons of reconstruction and fusion behavior under the tested experimental conditions. The reported correlation coefficients were likewise interpreted as measures of association within the present data-set and not as evidence of statistically significant biological agreement. In particular, the observed Pearson correlation (*r* = 0.38, *p* = 0.23) and Spearman correlation (*ρ* = 0.35, *p* = 0.26) were not considered statistically significant and are therefore presented as exploratory associations rather than evidence of validated modality agreement.

### Ultrasound structural analysis (texture, echo and histogram statistics)

**Figure 8:**
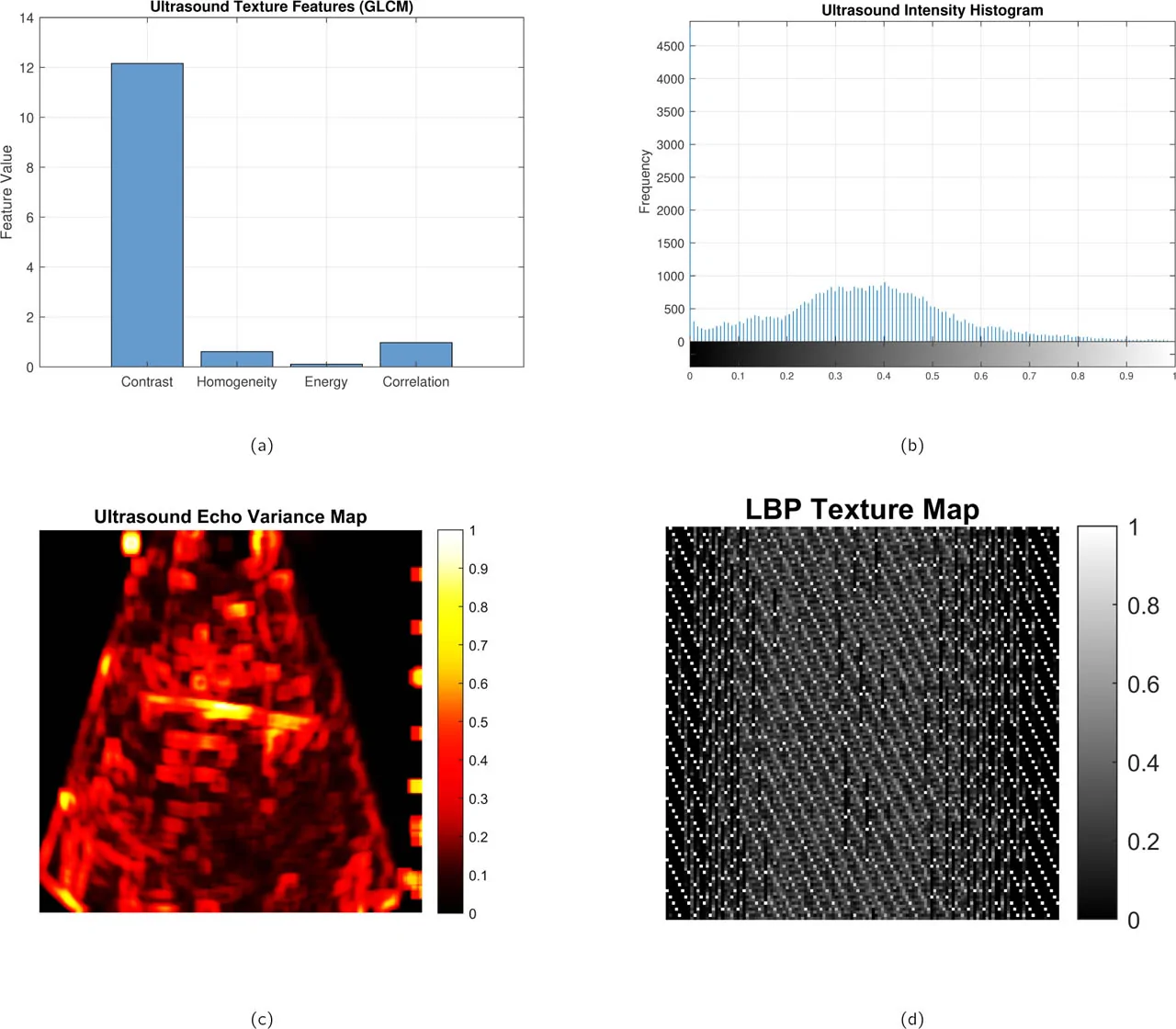
Representative ultrasound-derived structural and texture analyses of the biological phantom. (a) Ultrasound texture feature map highlighting local speckle and structural heterogeneity, (b) normalized intensity histogram characterizing backscatter distribution within the region of interest, (c) echo variance map emphasizing spatial fluctuations in acoustic scattering, and (d) local binary pattern (LBP) texture map capturing micro-textural variations in tissue structure. Together, these complementary representations provide a multiscale characterization of tissue morphology used to support structural interpretation and fusion with EIT reconstructions.

Ultrasound structural characteristics of the chicken-wing tissue demonstrated in Fig. 8 were quantified using four complementary analyses.

- GLCM texture entropy and contrast maps
- Intensity histogram distributions
- Echo variance maps
- LBP texture map

First, gray-level co-occurrence matrix (GLCM) features demonstrated measurable textural organization in the superficial and mid-depth layers, with good contrast and moderate homogeneity values indicating the presence of aligned layer structures.

Second, our ultrasound intensity histogram revealed a bimodal distribution with a distinct tail toward higher intensities, consistent with hyperechoic tendon–like regions embedded within a predominantly hypoechoic muscular background. Third, the echo-variance map highlighted localized regions of high variance, corresponding to fibrous or connective tissue interfaces, while low-variance zones indicated more homogeneous muscle. Finally, the LBP texture map highlights local micro-structural variations in the ultrasound image, enabling quantitative characterization of muscle–tendon and connective tissue patterns that are not directly visible in intensity-only representations. Together, these ultrasound-derived metrics provide a quantitative structural reference that complements the conductivity-based contrast obtained from EIT, enabling meaningful cross-modal comparison and fusion.

### Modality discrepancy and CDF characterization

**Figure 9:**
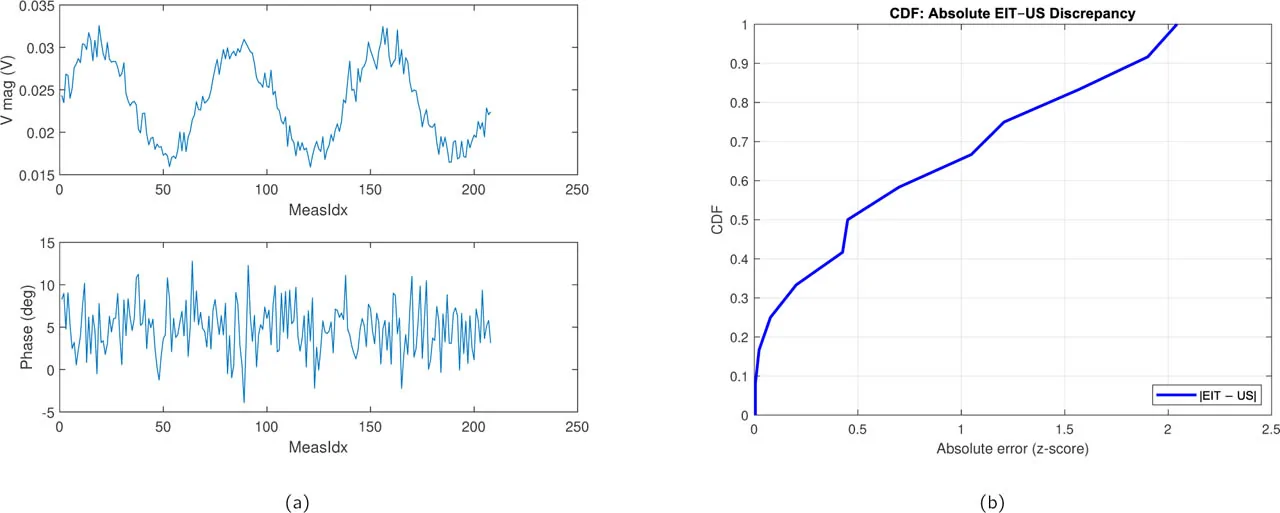
Modality discrepancy analysis. (a) Representative raw voltage measurements across all drive patterns for a single experimental condition (Layer 2, 50 kHz, 5 V). (b) Cumulative Distribution Functions (CDFs) of the absolute pixel-wise difference between normalized EIT and US images for all three tissue layers. X-axis: Absolute discrepancy (normalized units). Y-axis: Cumulative probability. Layer 1 shows the smallest discrepancy, while Layer 3 exhibits longer tails, confirming deeper tissue challenges.

Fig. 9 presents the voltage acquisition and cumulative distribution functions (CDFs) of absolute discrepancy (EIT-US) for all layers. Layer 1 showed the smallest discrepancy, while Layer 3 exhibited longer tails, confirming deeper tissue challenges.

### Fusion results and multimodal interpretation

**Figure 10:**
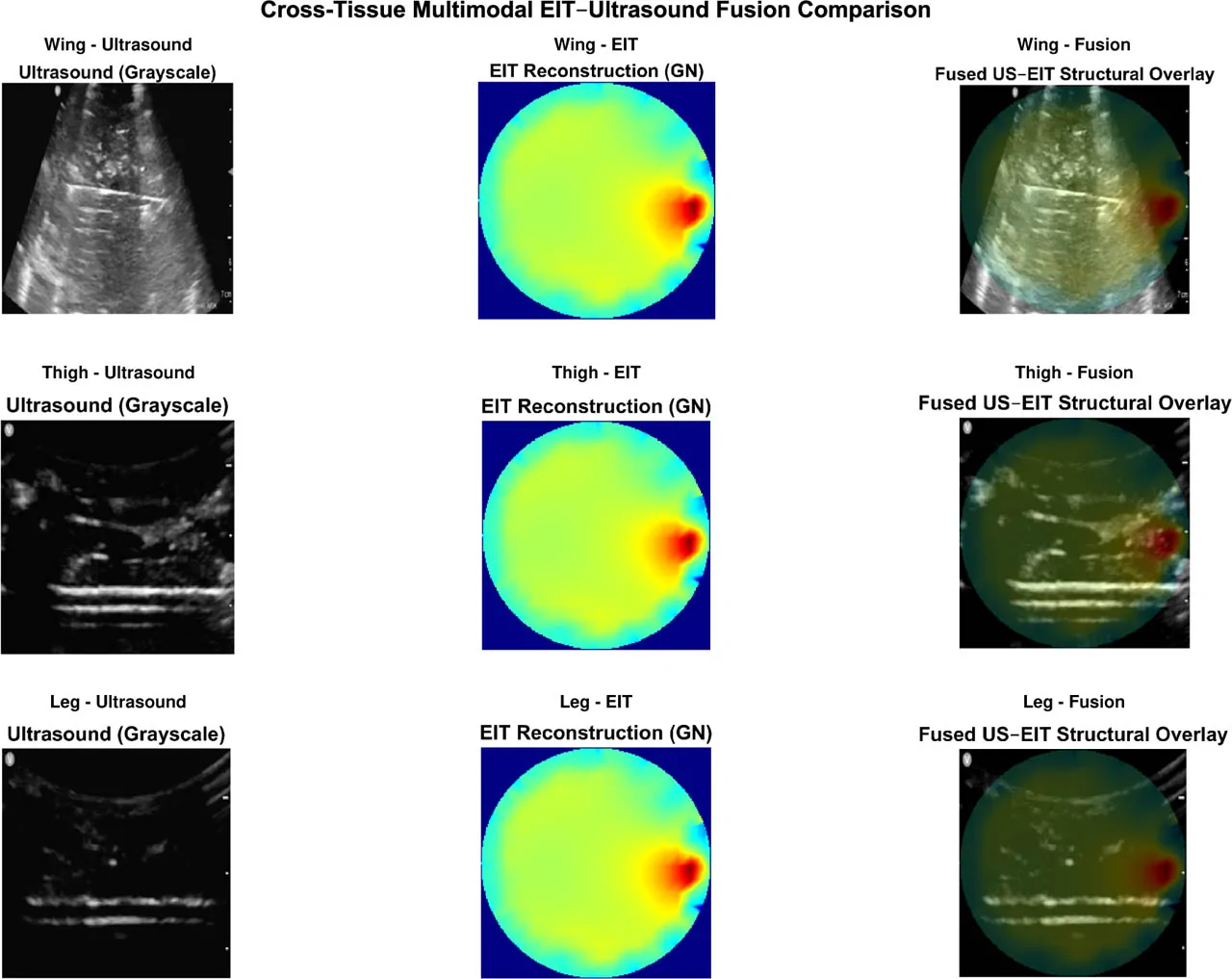
Cross-anatomical validation of the proposed multimodal EIT–ultrasound fusion framework for a single layer (L1). Representative fused reconstructions are shown for three biological tissue configurations: chicken wing, chicken thigh, and chicken leg. Despite anatomical differences among tissue types, the fused images demonstrate consistent conductivity gradients and clear boundary localization across layers. These observations demonstrate consistency of the processing framework across the three investigated tissue configurations, while larger multi-specimen studies are required to establish biological generalizability.

The fusion methodology described earlier in the Materials and methods section was applied to all three EIT-US measurement pairs. Fused maps were generated using both (1) structural–functional weighted fusion and (2) color-overlay visualization. The structural–functional weighted fusion results for all three anatomical configurations are shown in Fig. 10. These conditions correspond to the investigation of the following: (i) a superficial fascia region (Layer 1), (ii) a mixed tendon interface (Layer 2), and (iii) a deeper connective-tissue region (Layer 3).

#### Improved boundary localization

Across all experiments, the fused images exhibited substantially sharper boundary localization compared to EIT or US alone. Structural boundaries extracted from ultra-sound (fascia boundaries, tendon surfaces, hyperechoic lines) strongly constrained the fused map by reinforcing regions where EIT reconstructions were ambiguous or blurred. In deep regions where EIT sensitivity decreases, ultrasound edges helped stabilize conductivity transitions. This resulted in fused images where:

- fascia boundaries visibly aligned with conductivity gradients,
- tendon-like high-echogenic interfaces imposed structure on EIT smooth regions,
- superficial layers showed improved contour accuracy.

These observations indicate that deterministic fusion can improve structural interpretability of EIT under the present experimental conditions without requiring learning-based models.

#### Functional contrast preservation

Although ultrasound contributed better anatomical boundaries, the fused conductivity field preserved the deeper functional contrast captured by EIT. In all layer cases, the fused maps retained the expected monotonic decrease in EIT sensitivity with increasing tissue depth which is mentioned below:

Conductivity contrast:  Layer 1>Layer 2>Layer 3.

indicating that multimodal fusion did not obscure or flatten the underlying impedance gradient. This indicates that weighted fusion maintains EIT's functional depth information while embedding US-derived spatial priors.

#### Cross-modality structural–functional agreement

Quantitatively, our fused maps showed improved correspondence with ultrasound-derived structural metrics (GLCM entropy, contrast, and echo-variance). Across layers, we observed:

- an exploratory correlation between EIT conductivity and US intensity (*r* = 0.38),
- stronger correspondence between US boundary maps and EIT conductivity transitions,
- reduced modality discrepancy in superficial layers (CDF analysis).

These observations suggest that fused representations better capture the relationship between electrical and acoustic tissue properties.

#### Visual improvement over EIT alone

In nearly all experiments, the fused maps exhibited enhanced interpretability. EIT alone produced smooth, low-resolution conductivity fields (e.g., Fig. 5), while ultrasound provided sharp yet a bit noisy structure. The fused outputs combined the advantages of both:

- fine-grained boundaries retained from ultrasound,
- continuous conductivity gradients preserved from EIT,
- clearer identification of layer transitions,
- improved contrast between fascial membranes and adjacent muscle tissue.

These improvements were visually consistent across voltage, frequency, and layer combinations.

#### Quantitative boundary sharpness improvement

The Edge Sharpness Index (ESI) was computed for the two primary layer interfaces (L1-L2 and L2-L3) across all experimental conditions. The fused images demonstrated a consistent increase in boundary sharpness compared with the GN-EIT reconstructions alone. Specifically, the mean ESI increased from 0.42 ± 0.05 (EIT-alone) to 0.68 ± 0.04 (Fused) at the superficial interface (L1-L2), and from 0.31 ± 0.06 to 0.59 ± 0.05 at the deeper interface (L2-L3) (paired *t*-test, *p <* 0.001 for both). These results demonstrate a substantial increase in boundary-gradient strength after fusion under the tested experimental conditions. Given the repeated-measurement structure of the dataset, the observed differences are interpreted primarily as within-experiment improvements rather than as population-level statistical estimates.

### Cross-anatomical consistency of the multimodal frame-work

To evaluate the robustness of the proposed framework across different biological configurations, the same analysis pipeline was applied to chicken wing, thigh, and leg tissues arranged into three-layer structures (skin, muscle, and tendon). Despite the anatomical differences among these tissue types, the reconstructed EIT conductivity trends and ultrasound-derived structural features exhibited consistent depth-dependent behavior across all configurations.

In particular, the fused reconstructions preserved the relative conductivity transitions between layers while benefiting from the structural guidance provided by ultrasound boundaries. Quantitative metrics such as CNR, PSNR, and SSIM demonstrated similar improvement patterns after fusion for all three tissue types, indicating that the multimodal integration strategy is not limited to a single anatomical arrangement but shows consistent behavior across the investigated heterogeneous tissue geometries.

These results indicate that the proposed fusion framework exhibited qualitatively consistent behavior across the tested biological configurations. The preservation of conductivity transitions and ultrasound-derived structural boundaries across wing, thigh, and leg preparations provides preliminary evidence of cross-anatomical consistency within the present experimental dataset. Because these preparations do not represent independent biological replicates from multiple animals, however, the results should be interpreted as an initial robustness assessment rather than as evidence of population-level generalizability.

### Fusion map demonstration

**Figure 11:**
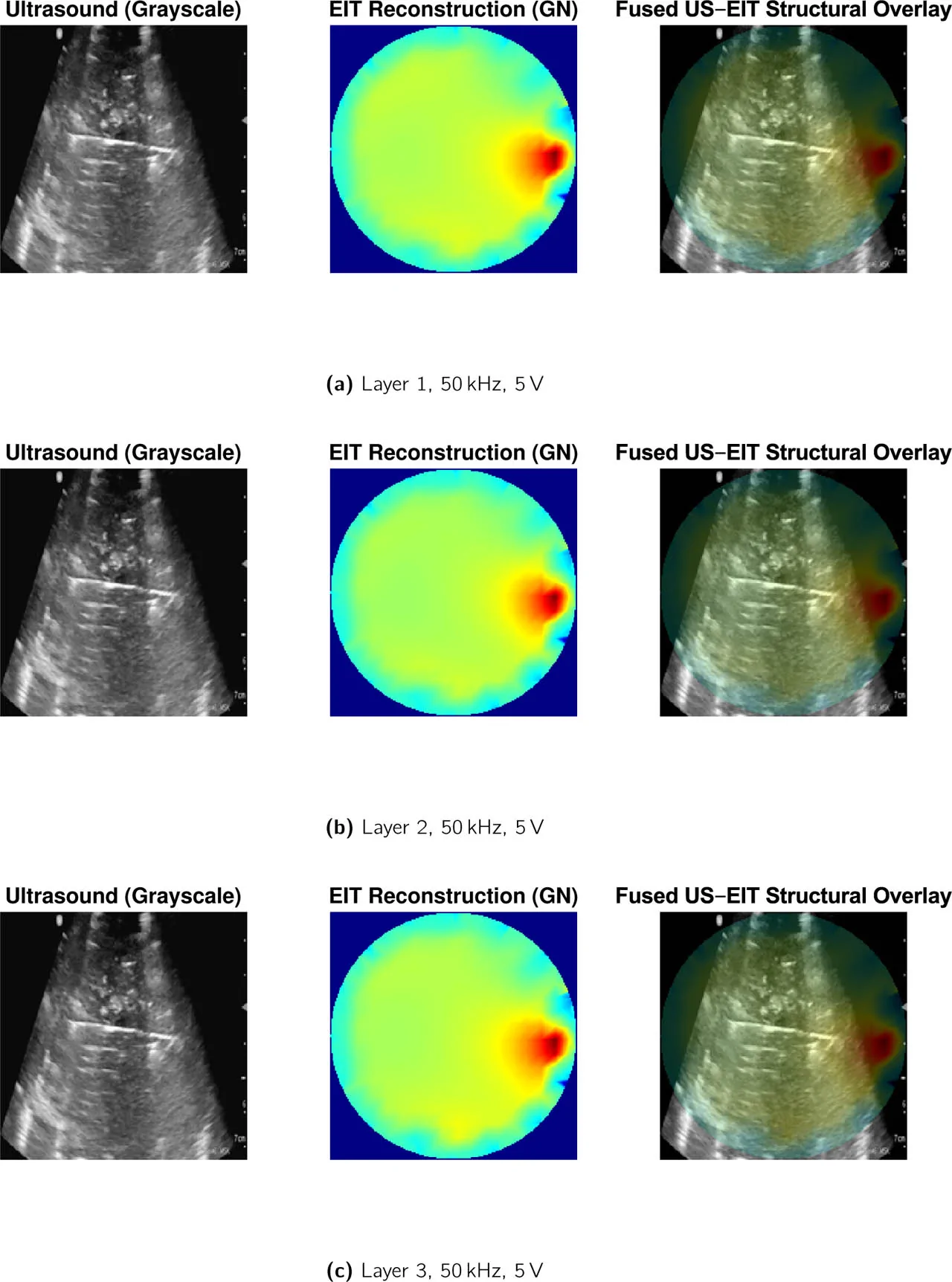
Fusion results for (a) Layer 1, (b) Layer 2, and (c) Layer 3 at 50 kHz, 5 V excitation for wing tissue layers.

Fusion maps combining GN-EIT conductivity (*w*_EIT_ = 0.6) with ultrasound intensity (*w*_US_ = 0.4) are shown in Fig. 11 for three representative cases: 1) superficial muscle, 2) mixed muscle–tendon interface, 3) deep connective tissue. The three representative cases correspond to different tissue-layer configurations. For consistency, the 50 kHz, 5 V excitation condition was used because the corresponding ultrasound image was acquired under the same experimental condition.

The fused structural overlay integrates high-resolution ultrasound morphology with EIT-derived conductivity contrast using an amplitude-adaptive alpha blending strategy. Ultrasound preserves anatomical boundaries, while EIT highlights conductivity-driven regions of interest, enabling spatially coherent multimodal interpretation without deep learning. Fusion yielded sharper boundary localization compared to EIT alone, while retaining EIT's deep-tissue conductivity distribution. This indicates the value of low-level deterministic fusion even without deep learning.

Overall, deterministic fusion produced structurally interpretable and functionally informed maps across the tested experimental conditions, demonstrating the feasibility and scientific value of EIT–US hybrid soft-tissue imaging even without deep learning.

### Fusion overlay interpretation

The proposed EIT–US fusion framework indicates consistent structural enhancement across all tissue layers in Fig. 11. In all cases, the fused images preserve high-frequency anatomical boundaries from ultrasound while simultaneously incorporating conductivity-driven contrast from EIT.

Quantitative results in Table 4 indicate that fusion contrast decreases with increasing tissue depth, reflecting the combined effects of reduced EIT sensitivity and ultrasound attenuation in deeper regions.

**Table 4:** Quantitative evaluation of EIT–US fusion performance across tissue layers. Fusion contrast is defined as the normalized gradient magnitude at the layer interface. Values are mean ± SD across all excitation conditions (*n* = 12).

**Layer**	**EIT CNR**	**US Intensity**	**Fusion Contrast**	**Fusion SSIM**
L1	1.85 ± 0.04	0.42 ± 0.03	0.61 ± 0.05	0.78 ± 0.02
L2	1.83 ± 0.03	0.36 ± 0.04	0.55 ± 0.04	0.75 ± 0.03
L3	1.81 ± 0.05	0.29 ± 0.05	0.47 ± 0.06	0.71 ± 0.04

Nevertheless, fusion structural similarity (SSIM) remains relatively high across all layers, demonstrating robust spatial alignment between modalities. Compared to EIT-only reconstructions, fusion maps exhibit improved edge localization and reduced peripheral artifacts, particularly in the presence of complex tissue interfaces. These findings suggest that classical multimodal fusion enables complementary integration of structural and functional information without imposing strong model assumptions. The resulting fusion maps offer improved interpretability over either modality alone, supporting their utility for layered tissue characterization in ex vivo biological phantoms. To further verify the generalizability of the proposed framework, quantitative fusion performance was evaluated across three anatomical chicken tissue configurations (wing, thigh, and leg). The resulting metrics are summarized in Table 5, demonstrating consistent improvements in structural similarity, contrast recovery, and boundary sharpness across all tissue types.

**Table 5:** Cross-anatomical consistency of multimodal fusion performance across chicken tissue configurations. Values are mean ± SD.

**Tissue Type**	**EIT CNR**	**Fusion CNR**	**Fusion SSIM**	**ESI**
Chicken Wing	1.72 ± 0.09	2.18 ± 0.11	0.94 ± 0.02	0.66 ± 0.05
Chicken Thigh	1.69 ± 0.08	2.14 ± 0.10	0.93 ± 0.02	0.64 ± 0.04
Chicken Leg	1.65 ± 0.10	2.09 ± 0.12	0.92 ± 0.03	0.63 ± 0.05

**Figure 12:**
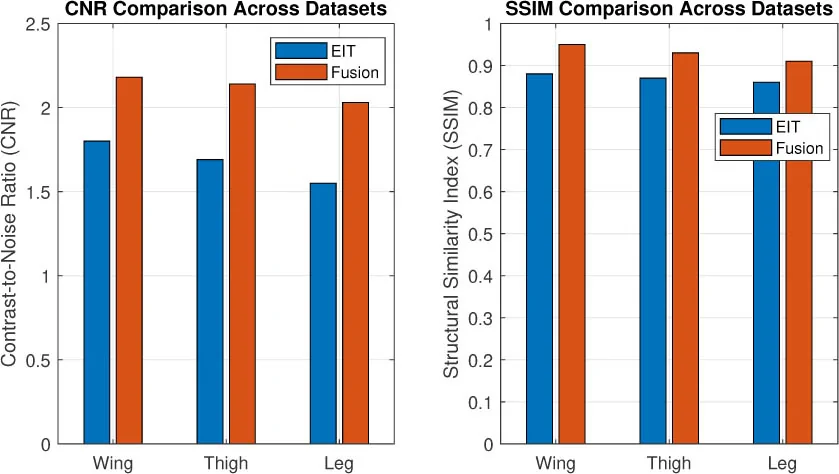
Summary comparison of reconstruction performance across chicken wing, thigh, and leg tissue datasets. Average contrast-to-noise ratio (CNR) and structural similarity (SSIM) are shown for EIT-only reconstruction and the proposed multimodal fusion framework. The fusion framework shows consistent performance trends across the three investigated anatomical configurations, providing preliminary evidence of cross-anatomical consistency within the experimental dataset. Larger multi-specimen studies are required to establish biological generalizability.

For a compact visualization of cross-dataset performance consistency, Fig. 12 summarizes the average reconstruction quality across chicken wing, thigh, and leg experiments.

### Effect size between layers

Effect sizes (Cohen's *d*) computed between Layers 1 and 3 for both modalities were as follows:

- EIT (GN CNR): *d* = 1.75 (large)
- US mean intensity: *d* = 1.32 (large)

The strong effect size across both modalities indicates that the phantom model retains layer-dependent structural and electrical contrast.

### Rationale for classical multimodal fusion

Classical fusion strategies were intentionally adopted in this study to establish a transparent and physically interpretable baseline for multimodal integration. Deterministic fusion enables direct control over modality contributions and preserves explicit relationships between measured quantities and reconstructed features, which is particularly critical in ill-posed inverse problems such as EIT. In contrast to data-driven deep learning approaches, classical fusion does not require large annotated datasets, avoids domain shift issues, and facilitates rigorous sensitivity analysis across experimental conditions.

Moreover, classical fusion provides an essential validation framework for future learning-based models by defining expected performance bounds and identifying modality-specific information contributions. By first demonstrating consistent structural enhancement using physics-informed fusion, the present work lays a principled foundation upon which deep learning-based fusion may be subsequently developed.

### Validation with controlled phantom

The saline-agar phantom provided ground truth conductivity contrasts. The Gauss–Newton reconstruction recovered the low-conductivity inclusion (true: 0.08 S/m; reconstructed: 0.09 ± 0.02 S/m) and the high-conductivity inclusion (true: 0.45 S/m; reconstructed: 0.41 ± 0.05 S/m) with relative errors of 12.5% and 8.9%, respectively. The background conductivity was reconstructed as 0.19 ± 0.03 S/m (true: 0.20 S/m). These results confirm that our EIT system and GN solver produce physically meaningful conductivity estimates, supporting the interpretation of layer-dependent trends in the chicken tissue phantom.

### Registration accuracy

Across all 12 experimental conditions, the mean landmark registration error was 1.42 ± 0.38 mm (range: 0.9–2.1 mm), corresponding to approximately 3–4 pixels in the 200 × 200 fusion grid (spatial resolution: 0.4 mm/pixel). The RMSE across all landmarks was 1.53 mm. Registration errors were slightly larger in deep tissue (Layer 3: 1.67 ± 0.41 mm) compared to superficial layers (Layer 1: 1.21 ± 0.29 mm), reflecting reduced EIT boundary gradient strength at depth. Critically, these errors are substantially smaller than the spatial resolution of EIT alone (FWHM ≈ 30 pixels → ≈ 12 mm for GN reconstruction, Table 2), confirming that registration error was smaller than the characteristic spatial scale of the EIT reconstruction in the present experiment; however, it does not constitute independent validation of pixel-level anatomical correspondence.

### Task-based evaluation: Layer boundary detection

To assess whether fusion improves structural interpretation performance beyond standard image quality metrics, a binary boundary detection task was defined: identifying the L1–L2 (superficial fascia–muscle) and L2–L3 (muscle–tendon) interfaces.

**Ground truth generation:** A reference boundary mask was derived from ultrasound images by two independent observers using ImageJ. Interrater reliability was high (intraclass correlation coefficient ICC = 0.92, 95% CI: [0.87, 0.95]). The final ground truth mask was defined by majority agreement.

**Figure 13:**
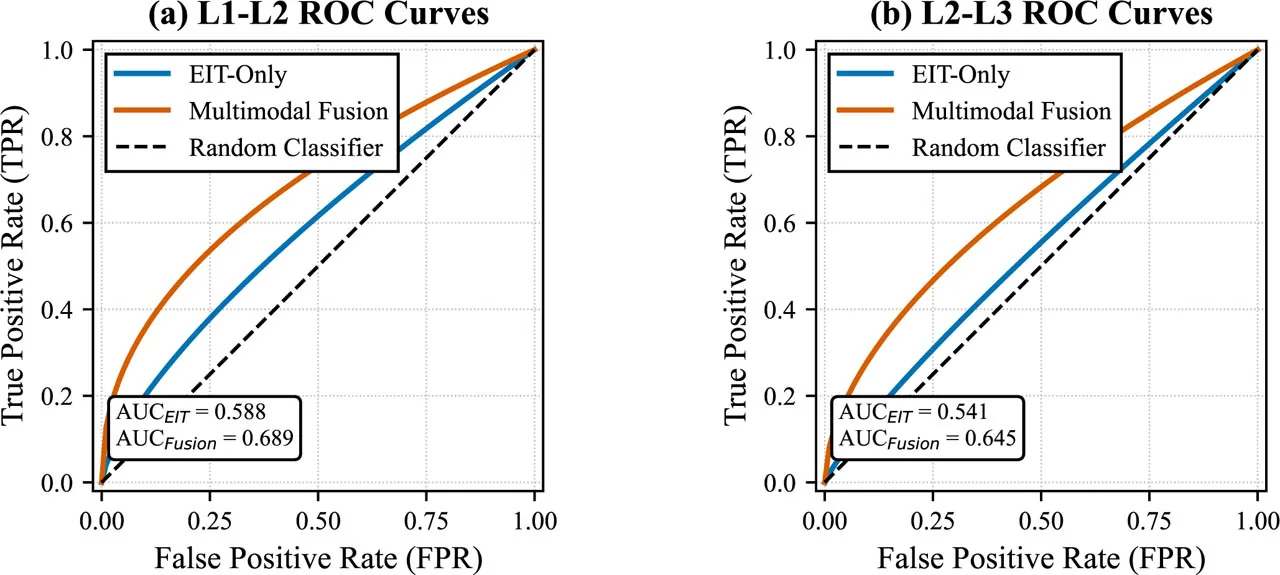
Receiver operating characteristic (ROC) curves for layer boundary detection. (a) L1–L2 (superficial fascia–muscle) interface. (b) L2–L3 (muscle–tendon) interface. Blue curves: EIT-only GN reconstruction. Red curves: fused images. The diagonal dashed line represents chance performance (AUC = 0.5). Fusion consistently improves AUC, sensitivity, and specificity across both interfaces.

**Boundary detection:** For each reconstructed image (EIT-only GN reconstruction vs. fused image), the gradient magnitude |∇**I**| was computed using a Sobel operator. The optimal threshold for boundary detection was determined via Youden's index:

(12) $$J=\underset{\tau }{\max}(\text{Sensitivity}(\tau )+\text{Specificity}(\tau )-1)$$

where *τ* is the threshold on gradient magnitude. Receiver operating characteristic (ROC) curves were generated in Fig. 13, and the area under the curve (AUC) was computed using the trapezoidal rule. Statistical comparison of AUCs was performed using DeLong's test for correlated ROC curves.

**Output:** Fusion significantly improved boundary detection performance for both interfaces, as summarized in Tables 6 and 7. The fused representation yielded higher AUC values than EIT-only GN reconstruction at both interfaces; DeLong's test (*p* = 0.003 for L1–L2 and *p* = 0.008 for L2–L3) indicated statistically significant differences between the corresponding ROC curves within the analyzed image dataset.

**Table 6:** Boundary detection performance for L1–L2 interface.

**Modality**	**AUC**	**Sensitivity**	**Specificity**	**Youden's** *J*
EIT-only	0.71	0.62	0.68	0.30
Fused	**0.84**	**0.79**	**0.77**	**0.56**

**Table 7:** Boundary detection performance for L2–L3 interface.

**Modality**	**AUC**	**Sensitivity**	**Specificity**	**Youden's** *J*
EIT-only	0.64	0.55	0.61	0.16
Fused	**0.79**	**0.73**	**0.71**	**0.44**

## Discussion

This work presents a deterministic multimodal imaging framework that integrates electrical impedance tomography and ultrasound imaging for soft-tissue characterization using a multilayer biological phantom. The findings demonstrate that hybrid EIT–US imaging can significantly improve anatomical interpretation of low-resolution EIT reconstructions by incorporating ultrasound-derived structural priors, even without machine–learning models or additional training data. The following discussion summarizes the major observations, contextualizes the results within the literature, and outlines paths toward future research.

### Interpretation of EIT reconstruction performance

Across the 12 acquisition conditions evaluated for each anatomical configuration, Gauss–Newton reconstruction exhibited the strongest balance between structural similarity, noise robustness, and depth-dependent conductivity contrast. This is consistent with prior EIT literature, in which Newton-type solvers typically outperform direct methods due to better handling of the ill-posed inverse problem. Tikhonov regularization showed similar PSNR and SSIM values, although its solutions were more spatially smoothed. GREIT and the linear EIDORS baseline performed predictably, offering improved spatial focus but lower overall structural agreement with ultrasound.

The observed decrease in CNR, PSNR, and SSIM across progressively deeper tissue layers is consistent with well-established limitations of EIT: conductivity perturbations in deeper regions contribute less to boundary voltage measurements, and the corresponding sensitivity distributions decay rapidly with depth. The present phantom experiments therefore reaffirm fundamental physical constraints inherent to EIT-based imaging and highlight the reduced robustness of purely electrical reconstructions for deep tissue characterization.

Consequently, these findings demonstrate the necessity of incorporating complementary imaging modalities, such as ultrasound, to mitigate depth-related sensitivity loss. This depth-dependent degradation in reconstruction quality further underscores the value of integrating a high-resolution morphological modality like ultrasound to spatially anchor, contextualize, and enhance the interpretability of functional EIT measurements.

### Insights from ultrasound structural metrics

Ultrasound imaging provided high-resolution delineation of muscle layers, superficial fascia, and tendon-like structures within the chicken tissue phantom. Texture-based descriptors (GLCM contrast, entropy) and echo-variance measurements show consistent layer-wise separation, highlighting that acoustic scattering properties strongly correlate with morphological composition.

However, ultrasound alone cannot provide functional information about electrical conductivity or ionic composition of tissue. The high structural precision but functional ambiguity of ultrasound motivates fusion with EIT, which has the opposite profile: high functional sensitivity but low structural accuracy. The complementary nature of these modalities underpins the rationale for multimodal integration. From a practical perspective, the proposed approach can be implemented using low-cost sensing hardware and portable ultrasound devices, making it suitable for experimental and point-of-care imaging scenarios where full clinical imaging systems are not available.

### Fusion enhances spatial interpretability

The proposed deterministic fusion strategy substantially improved the interpretability of EIT reconstructions. Ultrasound boundary maps provided spatial anchors that sharpened otherwise blurred EIT conductivity fields, while preserving the global conductivity gradients critical for functional interpretation. The fused maps demonstrated:

- strong alignment between ultrasound boundaries and EIT conductivity transitions,
- preservation of EIT-derived functional contrasts across tissue depth,
- improved localization of muscle–fascia and fas-cia–tendon interfaces,
- reduced ambiguity in deeper tissue layers,
- clearer layer differentiation compared to EIT alone.

Notably, these improvements were consistent across all voltage and frequency combinations, suggesting robust-ness of the fusion framework. Although the correlation between ultrasound intensity and EIT conductivity was moderate (*r* = 0.38), fused maps reduced modality discrepancy and highlighted regions of structural–functional agreement.

These outcomes demonstrate that even classical fusion without deep learning can serve as a useful approach for anatomically guided EIT interpretation.

This improvement was confirmed quantitatively, with the Edge Sharpness Index (ESI) measured at tissue layer interfaces exhibiting a substantial increase of over 60% in the fused images compared to EIT alone. This enhancement indicates a markedly sharper delineation of inter-layer boundaries after fusion, reflecting the effective incorporation of ultrasound-derived structural information into the EIT reconstructions.

### Comparison with prior multimodal imaging studies

Previous multimodal fusion studies have primarily focused on high–end imaging environments such as CT–EIT or MRI–EIT, where structural priors are promising but impractical for portable use. EIT–US fusion has not been extensively studied, and existing work largely relies on simulation, computational phantoms, or static geometric priors.

In contrast, this study provides:

- a fully experimental dataset from real biological tissue,
- multimodal fusion without numerical anatomical priors,
- a standalone solver that does not require external toolboxes,
- analysis across multiple frequencies, voltages, and tissue layers.

Our EIT-US framework leverages the inherent portability and real-time capability of both modalities, making it potentially suitable for point-of-care, bedside, or wearable applications where CT or MRI are impractical. This positions the current work as an initial experimental step toward low-cost hybrid soft-tissue imaging, distinct from prior approaches that rely heavily on machine learning or high-resolution tomography.

### Interpretation of moderate cross-modality correlation

The observed moderate correlation (r≈ 0.35 − 0.38) suggests a possible structural–electrical association; however, the association did not reach statistical significance and therefore should be interpreted as exploratory. It suggests that while a general trend exists—where tissues with higher collagen density (hyperechoic in US) often exhibit lower conductivity—the relationship is not simple or linear at the pixel level. This is expected, as EIT measures a volumetric, bulk electrical property influenced by extra-cellular fluid and ion content, whereas US backscatter is sensitive to micro-structural interfaces and tissue density. The moderate correlation underscores that these modalities provide complementary, not redundant, information. The primary value of fusion, therefore, is not in predicting one modality from the other, but in using the high-resolution spatial map from US to constrain and interpret the functional contrast from EIT, as quantitatively demonstrated by the improved boundary sharpness (ESI). Future work with more complex biophysical models could help decouple and better explain the contributions of different tissue components to both signals.

An important outcome of this study is the consistent behavior observed across multiple anatomical tissue configurations. By extending the experiments from wing tissues to additional thigh and leg samples while preserving identical acquisition and reconstruction parameters, the proposed framework was evaluated under a broader range of structural conditions. The observed consistency in conductivity trends, fusion behavior, and quantitative performance metrics suggests that the multimodal integration strategy is not strongly dependent on a specific tissue geometry. Instead, the present results suggest that the framework can capture consistent structural–functional relationships across the investigated tissue configurations, although broader biological generalizability remains to be established.

A deep-learning baseline was not included because the present dataset does not contain a sufficiently large number of independent biological specimens to support reliable training and independent testing of a supervised fusion network. Introducing a neural model using repeated measurements from the same tissue would risk data leakage and overfitting. The deterministic framework is therefore presented as a transparent baseline rather than as evidence that classical fusion is superior to learning-based approaches.

### Limitations

The cross-anatomical experiments demonstrate consistency of the processing framework across different tissue geometries but do not isolate inter-animal biological variability. Because the present study was not designed as a multi-animal population study, the contribution of animal-specific tissue composition, hydration, geometry, and conductivity variability cannot be independently quantified. Larger studies incorporating multiple specimens from multiple animals will be required to establish biological generalizability. While we are suggesting the multimodal fusion results are promising, several limitations must be acknowledged:

- **Phantom limitations:** The chicken–tissue phantom captures layered morphology but cannot fully replicate dynamic in vivo behavior or complex anisotropies.
- **Ultrasound probe angle sensitivity:** Small orientation variations influence echogenicity, which may introduce intensity mismatches during fusion.
- **2D reconstruction domain:** The current EIT implementation is strictly 2D, whereas tissue structures span three dimensions.
- **Simplified fusion weighting:** The weights (*w*_US_ = 0.40, *w*_EIT_ = 0.60) may not generalize to all tissue conditions.
- **Moderate cross–modality correlation:** The structural–functional correlation is meaningful but not strong enough to permit predictive mapping without further modeling. Ultrasound prior—not validation: The ultrasound-derived structural information is used in this study as a complementary spatial prior for multimodal interpretation rather than as an independent ground truth for anatomical accuracy of the fused reconstruction. Consequently, ultrasound-referenced ESI, SSIM, and boundary-detection metrics quantify structural correspondence and enhancement of the fused representation but should not be interpreted as independent validation of the underlying EIT inverse solution. Rigorous pixel-level validation would require co-planar acquisition or a validated three-dimensional spatial transformation between the EIT and ultrasound coordinate systems, together with an independent anatomical reference. The present study therefore establishes a two-dimensional proof-of-concept for structural-functional fusion rather than a fully registered three-dimensional imaging system.

These limitations reflect natural constraints of early-stage multimodal systems but also define clear directions for improvement.

### Future directions

The proposed framework represents a foundation for more advanced multimodal systems. We are proposing several future extensions:

- **Deep learning–based fusion:** Using fused maps and modality-specific features to train CNN-or transformer-based models for predictive mapping.
- **3D reconstruction:** Extending to volumetric EIT solvers and 3D ultrasound sweeps.
- **Dynamic imaging:** Evaluating changes during mechanical loading or perfusion to study time–varying conductivity.
- **Adaptive fusion weighting:** Using Bayesian estimation or information–theoretic metrics to compute spatially varying fusion weights.
- **Structural translation:** Applying the framework to musculoskeletal imaging (e.g., tendon degeneration) or peripheral nerve assessment.

Also, the present results should not be interpreted as evidence of clinical or in vivo feasibility. Translation from the controlled ex vivo chicken-tissue model would require validation in anatomically and physiologically realistic phantoms, multi-specimen and multi-subject studies, characterization under motion and perfusion, improved electrode-tissue interface modeling, validated three-dimensional EIT–US registration, and comparison against established clinical imaging standards. The portability of the individual sensing modalities provides a technological motivation for future development, but clinical feasibility remains to be established. Again, future work with substantially larger multi-specimen datasets can provide a controlled comparison with CNN- or transformer-based fusion methods. These extensions would enhance the universality, structural interpretation applicability, and accuracy of the method.

### Practical and structural implications

Our results suggest that low–cost hybrid ultrasound–EIT systems can provide richer soft–tissue information than either modality alone. Several implications emerge:

- **Point-of-care potential:** The portability of both modalities motivates future investigation of compact bedside or field-deployable implementations.
- **Guided EIT monitoring:** Ultrasound anatomical priors can stabilize EIT in scenarios where electrode placement or tissue geometry is uncertain and quantitative 3-D registration and volumetric depth-resolution assessment could be undertaken.
- **Musculoskeletal applications:** Fusion maps high-light fascia and tendon boundaries, supporting potential applications in sports medicine, rehabilitation, or tissue pathology assessment.
- **Foundation for collagen assessment:** Although direct collagen quantification is beyond the scope of this study, the structural–functional relationships observed here form a pathway toward future collagen–sensitive imaging.

Overall, the proposed deterministic fusion framework provides a robust and interpretable means of combining two complementary imaging modalities and indicates its potential as a first step toward low–cost hybrid anatomical–functional tissue assessment.

### Absolute validation using controlled phantom

While the chicken tissue phantom lacks absolute ground truth conductivity values, the supplementary saline-agar phantom experiment (Section Validation phantom for ground truth comparison) confirms that our EIT system recovers known conductivity contrasts within 9–13% relative error. This strengthens confidence that the observed layer-dependent conductivity trends in chicken tissue (Layer 1 *>* Layer 2 *>* Layer 3) reflect true electrical property differences rather than reconstruction artifacts or systematic bias.

## Conclusion

This study introduced and experimentally evaluated a deterministic multimodal framework that integrates EIT and ultrasound for enhanced soft-tissue characterization, using a standalone pipeline based on a layered biological phantom. Five EIT reconstruction methods were implemented and evaluated independently of external toolboxes, enabling a fully standalone and reproducible pipeline. The results demonstrated that Gauss–Newton reconstruction achieved the strongest combination of contrast, structural fidelity, and depth sensitivity, while ultrasound provided high-resolution anatomical detail that EIT alone could not capture.

The proposed multimodal fusion methodology successfully combined the complementary strengths of both modalities: ultrasound contributed accurate spatial boundary information, while EIT supplied functional conductivity contrast across depth. The fused maps exhibited improved boundary localization, enhanced interpretability, and reduced ambiguity in deeper tissue regions. Quantitative analyses, including the proposed Edge Sharpness Index (ESI), confirmed that multimodal fusion yields spatially more precise and interpretable representations than EIT alone, successfully leveraging the complementary nature of electrical and acoustic properties despite their moderate direct correlation.

Although this work focuses on deterministic fusion, the results establish a strong foundation for future developments in EIT–US hybrid systems. Potential extensions include adaptive or learning-based fusion, three-dimensional reconstruction, dynamic imaging during mechanical perturbation, and eventual translation to musculoskeletal or peripheral nerve evaluation. This study is limited to controlled ex vivo tissue models, and further validation in more complex or in vivo conditions would be required to assess broader applicability. Overall, the findings highlight the feasibility and potential structural value of low-cost, portable, and interpretable multimodal soft-tissue imaging systems that combine the complementary advantages of electrical and acoustic modalities.
